# Systematic Review of Management of Moderate Wasting in Children over 6 Months of Age

**DOI:** 10.3390/nu15173781

**Published:** 2023-08-30

**Authors:** Zahra A. Padhani, Bernardette Cichon, Jai K. Das, Rehana A. Salam, Heather C. Stobaugh, Muzna Mughal, Alexandra Rutishauser-Perera, Robert E. Black, Zulfiqar A. Bhutta

**Affiliations:** 1 Robinson Research Institute, Adelaide Medical School, University of Adelaide, Adelaide, SA 5000, Australia; 2 Institute for Global Health and Development, Aga Khan University, Karachi 74800, Pakistan; jai.das@aku.edu (J.K.D.); or zulfiqar.bhutta@sickkids.ca (Z.A.B.); 3 Action against Hunger UK, London SE10 0ER, UK; muznamughal15@gmail.com (M.M.); a.rutishauserperera@actionagainsthunger.org.uk (A.R.-P.); 4 Division of Women and Child Health, Aga Khan University, Karachi 74800, Pakistan; 5 Centre of Research Excellence, Melanoma Institute Australia, University of Sydney, Sydney, NSW 2006, Australia; asalam.rehana@gmail.com; 6 Action against Hunger USA, Technical Services and Innovation Department, Washington, DC 20463, USA; hstobaugh@actionagainsthunger.org; 7 Friedman School of Nutrition Science and Policy, Tufts University, Boston, MA 02111, USA; 8 Bloomberg School of Public Health, Johns Hopkins University, Baltimore, MD 21205, USA; rblack1@jhu.edu; 9 Centre for Global Child Health, Hospital for Sick Children, Toronto, ON M5G 0A4, Canada

**Keywords:** moderate wasting, moderate acute malnutrition, specialized formulated foods, fortified-blended foods (FBF), ready-to use supplementary foods (RUSF), nutrition counselling

## Abstract

The effective management of the 33 million children with moderate acute malnutrition (MAM) is key to reducing childhood morbidity and mortality. In this review, we aim to evaluate the effectiveness of specially formulated foods (SFFs) compared to non-food-based approaches to manage MAM in children >6 months old. We conducted a search on ten databases until 23 August 2021 and included five studies, covering 3387 participants. Meta-analysis of four studies comparing SFFs to counselling or standard of care showed that SFFs likely increase recovery rate, reduce non-response, and may improve weight-for-height z-score, weight-for-age z-score and time to recovery, but have little or no effect on MUAC gain. One study on a multicomponent intervention (SFFs, antibiotics and counselling provided to high-risk MAM) compared to counselling only was reported narratively. The intervention may increase weight gain after 24 weeks but may have little or no effect on weight gain after 12 weeks and on non-response and mortality after 12 and 24 weeks of enrollment. The effect of this intervention on recovery was uncertain. In conclusion, SFFs may be beneficial for children with moderate wasting in humanitarian contexts. Programmatic recommendations should consider context and cost-effectiveness.

## 1. Introduction

Children who suffer from moderate wasting (also referred to as moderate acute malnutrition (MAM), which is characterized as a weight-for-height z-score (WHZ) < −2 and ≥−3 or a mid-upper arm circumference (MUAC) < 125 mm and ≥115 mm in children 6–59 months), face a threefold higher risk of mortality as compared to adequately nourished children [1].

If not treated, the condition can easily lead to severe acute malnutrition (SAM), which entails an even more pronounced risk of near-term mortality [1]. Those that survive experience adverse health effects and negative developmental outcomes. Recent prevalence estimates suggest that in 2020, nearly 31 million children <5 years of age had moderate wasting [2]. The actual number of children impacted by MAM every year is likely much larger than this estimate because many factors, such as the incidence of moderate wasting and MUAC-based case definitions, are not considered in this figure [3,4,5].

The effective management of children with MAM is therefore key to reducing childhood morbidity and mortality, and to reaching the World Health Assembly (WHA) targets of lowering wasting prevalence to below five percent by 2025 and less than three percent by 2030 [6]. Until very recently, the World Health Organization (WHO) had no guidelines for the management of MAM, but the Essential Nutrition Actions encompassed some guidance based on breastfeeding promotion and support and nutrition education or counselling. Furthermore, in humanitarian emergencies where the increased nutrient and energy requirements for moderately wasted children cannot be met through locally available foods, specially formulated foods can be provided. Such foods typically take the form of fortified blended foods (FBFs) or ready-to-use supplementary foods (RUSFs) that are provided as part of outpatient-based, supplementary feeding programs [7,8,9].

Overall, the practice for managing MAM differs by location, national-level guidance, and implementing organization. More recently, innovations and adaptations in the management of acute malnutrition, including MAM, have been implemented with aims to improve efficiency in operations, maximize coverage and reduce overall costs. The types of program adaptations vary, but many include reducing the frequency of clinic-based follow-up visits, training caregivers to identify acute malnutrition in their own children, treating children closer to the communities through community health workers, simplifying admission criteria, and/or combining the treatment of SAM and MAM in one program [10,11,12].

In 2020, WHO initiated the development of a comprehensive guideline focused on the prevention and management of acute malnutrition in infants and children. This guideline now incorporates recommendations for the treatment of MAM for the first time. As part of this, the WHO commissioned two systematic reviews on the management of moderate wasting. The first review explored the effectiveness of different types, quantities and durations of dietary management with specially formulated foods (SFFs) and has recently been published [13]. The aim of this subsequent review was to evaluate the effectiveness of food-based approaches compared to other interventions with or without non-specially formulated food to manage MAM in infants and children over six months of age. Furthermore, an aim was to identify which subgroups of children are most likely to benefit from SFFs.

## 2. Materials and Methods

### 2.1. Inclusion and Exclusion Criteria

The inclusion and exclusion criteria have been briefly outlined in Table 1 using the Participant, Intervention, Comparators, Outcomes and Study Design (PICOS) criteria.

The review included studies evaluating the impact of food-based approaches to manage MAM. Studies including wider definitions of undernutrition were only included if the results were presented separately for moderately wasted children based on at least one of the criteria, either MUAC or WHZ. Specifically, studies assessing the following comparisons were included:

*Comparison 1:* All food-based vs. non-food-based approaches or none.

*Comparison 2:* Specially formulated foods vs. non-food-based approaches or none.

*Comparison 3:* Specially formulated foods vs. non-specially formulated foods.

*Comparison 4.* Multicomponent interventions vs. standard of care or none.

**Figure 1:**
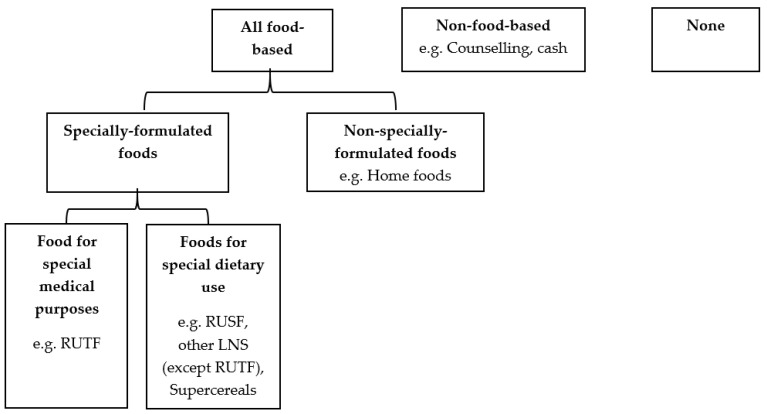
Flow chart of intervention categories.

The intervention categories (Figure 1) are based on the inputs involved in each approach. All food-based approaches include the provision of any type of food, regardless of its composition or originally intended use. Non-food-based approaches include any inputs other than direct food distribution, such as cash or counselling. While counselling often includes instructions for caregivers to provide additional foods and specific types of foods to their malnourished children, the food itself is not directly provided as an input of the program or health service. Therefore, counselling is considered a non-food-based approach. No intervention (labelled as “none” above) is defined as providing either inputs, assistance, or services of any kind.

SFFs for the treatment of MAM are characterized as foods designed, produced, distributed, and utilized in at least one of two distinct ways: (1) foods tailored for specific dietary requirements; or (2) foods intended for particular medical purposes as outlined by the Codex Alimentarius for International Foods [14,15]. In this review, foods classified as “for special dietary uses” pertain to those that can be widely distributed without the need for medical supervision, e.g., RUSF, FBFs, or other diverse forms of lipid-based supplements (LNS). These foods are also frequently used in the management of MAM, but may also have other uses. Foods “for special medical purposes” are intended to be distributed with medical oversight, e.g., ready-to-use-therapeutic food (RUTF). Non-specially formulated foods, often called “local foods” or “home foods”, refer to the foods that are commonly consumed by the population without being specifically designed to address malnutrition. This may include imported foods, foods grown locally, or provision of food by development partners or humanitarian organizations in response to food scarcity. Multicomponent interventions are defined as those including any combination of the above categories. Such interventions were included, but analyzed separately.

### 2.2. Search Strategy and Study Selection

An extensive search was conducted by formulating a search using the PICO criteria as described in Table 1, and the full search strategy can be found in Supporting Table S1. Search was conducted on 10 electronic databases including Medline/PubMed, Embase, Web of Science Index Medicus, CINAHL, Lilacs, the Cochrane Central Register of Controlled Trials (CENTRAL), and eLENA (WHO), Index Medicus for the WHO Eastern Mediterranean Region, and African Index Medicus. No restriction was applied on language or date. The date of the final search was 23 August 2021. We cross-referenced the bibliographies of all included studies, relevant systematic reviews and scoping review on MAM conducted for WHO by this research group in November 2020, to identify studies that may have been missed in the initial search.

Studies identified through database search were exported into EndNote and underwent deduplication. The records were then uploaded into Covidence [16] software for title/abstract and full-text screening. Two reviewers independently screened papers, at both the title/abstract and full-text screening stage, to determine relevance based on the aforementioned selection criteria. Discrepancies were resolved through discussion or by contacting a third reviewer who was a subject expert.

### 2.3. Data Extraction

Data were extracted from the included studies by two independent reviewers into an Excel spreadsheet. Discrepancies were resolved through discussion or by consulting a third reviewer if consensus was not achieved. Data were extracted on the following: study setting and methods, participants characteristics, inclusion/exclusion criteria, intervention/comparison characteristics, outcomes of interest (at baseline and endline), follow-up details, and additional information such as study limitations, funding, and conflict of interest.

### 2.4. Risk of Bias (ROB) Assessment

Two authors conducted individual assessments of the quality of all eligible studies using the updated Cochrane risks of bias tool, ROB-2 [17]. Any differences in evaluations were resolved through consensus or by consulting a third author.

### 2.5. Data Analysis

Analysis was conducted on Review Manager (RevMan) 5.4 software [18]. All the estimates were verified by a second author. Dichotomous outcomes are presented as risk ratios (RR), while the continuous outcomes are presented as mean difference (MD) or standardized mean difference (SMD) along with 95% confidence intervals (CI). Data were analyzed separately for the four comparisons, as mentioned above.

In the comparison of SFFs versus non-food-based or none (Comparison 2), the non-food-based category encompassed a diverse range of intervention types. Nevertheless, a meta-analysis was conducted as the studies fit into this comparison. A separate analysis was conducted for single and multicomponent interventions. All cRCTs were adjusted for clustering so no further adjustment of clusters was deemed necessary during the analysis.

We considered creating funnel plots to investigate potential biases related to small studies and publication biases. However, this was not possible due to the limited number of studies available under each outcome.

### 2.6. Subgroup and Sensitivity Analysis

Subgroup analyses based on study context, age group, duration of intervention, type of dietary intervention or comparison group, type and dosage of specially formulated food, facility vs. community-based approaches, HIV status and morbidity, concurrent stunting, breastfeeding status, household socio-economic status as well as means of identification (routine screening vs. presentation to a facility) were planned. We were able to perform subgroup analysis based on the type of comparison group for SFFs comparison only; however, we were unable to perform subgroup analysis based on other criteria because of very few studies being included in the review and due to insufficient information being available. We performed sensitivity analysis on the outcomes to assess the impact of high/unclear risk of bias relating to sequence generation and allocation concealment.

Furthermore, a subgroup analysis was planned to identify which children require SFFs, across settings and contexts, but unfortunately this was not possible given the small number of studies identified.

### 2.7. Evidence Profiles

We constructed GRADE evidence profiles for primary outcomes, summarizing the quality of evidence in accordance to the Grading of Recommendations, Assessment, Development, and Evaluation (GRADE) criteria [19]. It encompasses evaluating factors including within-study risk of bias, indirectness, inconsistency, imprecision, and risk of publication bias. The certainty of evidence for each outcome was rated as “very low” “low”, “moderate” or “high”.

Preferred Reporting Items for Systematic reviews and Meta-Analyses (PRISMA) guideline was followed for reporting (See Supporting Table S2). The review protocol was registered with the International Prospective Register of Systematic Reviews (PROSPERO: CRD42021273394).

## 3. Results

### 3.1. Search Results

**Figure 2:**
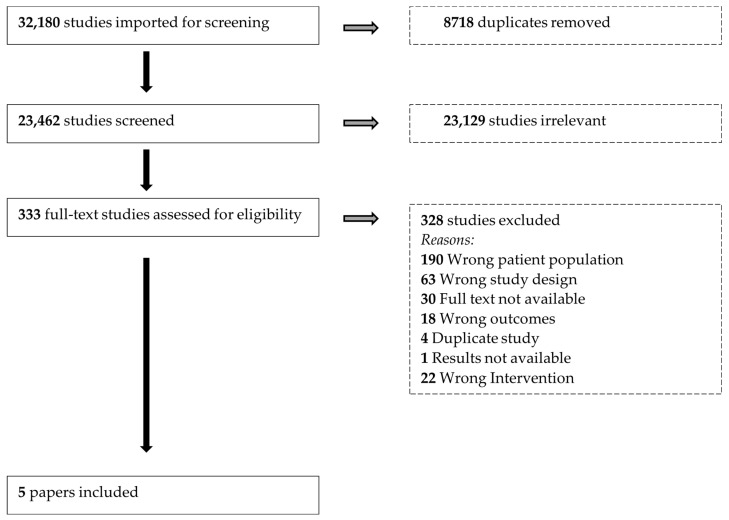
PRISMA flow diagram.

We identified a total of 32,180 records for screening. After de-duplication, 23,462 records underwent title and abstract, followed by full-text screening. A total of five papers was included for data extraction and analysis (Figure 2).

**Figure 3:**
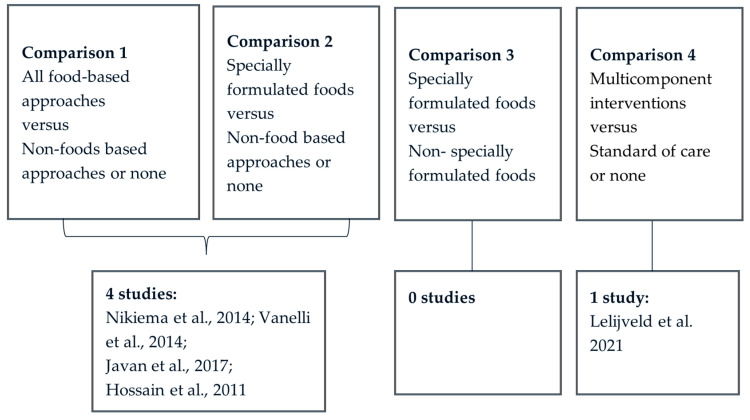
Studies identified by comparison group [20,21,22,23,24].

Figure 3 outlines the number of studies included by comparison group. Four studies fit into the first two comparisons. Specially-formulated foods is a subgroup of all food-based approaches, therefore any studies fitting into comparison 2 were also included in comparison 1 (Figure 1). All four studies identified for these two comparisons included interventions using SFFs, including either foods for special medical purposes or special dietary uses. We did not identify any study that looked at non-specially formulated foods only. Therefore, there were no differences in the number of studies between comparison 1 and comparison 2. Going forward in this paper, we will refer to comparison 2 for these included studies.

### 3.2. Characteristics of Included Studies

Five studies, including 3387 participants, were included in this review [20,21,22,23,24]. Studies were conducted between 2011 and 2021 in Burkina Faso [22], Sierra Leone [23], Malawi [21], Iran [20] and Bangladesh [24]. Out of the five studies, three were individually randomized RCTs [20,23,24] and two were cluster randomized RCTs [21,22].

#### 3.2.1. Comparison 2: Specially-Formulated Foods vs. Non-Food-Based or None

A total of four studies fit into comparison 2, namely the studies by Hossain et al., 2011 [24], Nikièma et al., 2014 [22], Javan et al., 2017 [20] and Vanelli et al. [23]. Table 2 provides a description of these four studies. The studies differed greatly in terms of study design, types of interventions, admission and discharge criteria for treatment, types of SFFs, and dosing of those formulated foods also differed greatly (Table 2). One study used a dose of 150 kcal/day for children < 12 months and 300 kcal/day for children 12–24 months [24], two studies used a dose of approximately 250 kcal/day [20,22], one study used a much higher dose of 200 kcal/kg/day in addition to a food ration providing 1000–1200 kcal/child/day [23].

#### 3.2.2. Comparison 4: Multicomponent Intervention versus Non-Food-Based or None

One study [21] was a multicomponent intervention. In this study by Lelijveld et al., children identified as high-risk MAM received a combination of 520 kcal/day of RUTF, amoxicillin, and nutrition counselling in the intervention group, while low risk MAM children received counselling only.

In the control group, both high-risk and low-risk MAM children received counselling only. In this review, we included only the high-risk subsample given that the low-risk group received counselling in each group and therefore did not meet the PICOS criteria. High-risk MAM children were those with at least one of the following characteristics: MUAC < 11.9, weight for age z-score (WAZ) < −3.5, primary caregiver is not the mother, and non-breastfed if the child is under 2 years. These characteristics have been associated with failure to recover in some Targeted Supplementary Feeding Program (TSFP) in Sierra Leone; thus, “high-risk” equates to failing to respond to the treatment provided in a TSFP.

### 3.3. Quality of Included Studies

Out of five studies, four had some methodological concerns related to the randomization process, deviation from the intended interventions, and selection of the reported result and one study was judged to be at high risk of bias (Supporting Figure S1).

### 3.4. Effects of Intervention

Four out of the five studies fit into comparison 1 and 2 with 2677 participants; and one study fit into comparison 4 (multicomponent intervention vs. standard of care) including 710 children. The same four studies were identified for comparison 1 and comparison 2 as all included interventions using SFFs and specially-formulated foods is a subgroup of all food-based approaches (Figure 1). No additional studies were identified that fit only the broader definition of interventions in comparison 1’s “all food-based approaches” category.

The results in terms of effect of intervention in comparison 1 and 2 and comparison 4 are presented below. One of the main objectives of this review was to carry out a subgroup analysis to determine which children might need supplementary foods; unfortunately, due to the limited data available, this was not possible.

#### 3.4.1. Comparisons 1 and 2: Specially Formulated Foods versus Non-Food-Based or None

**Figure 4:**
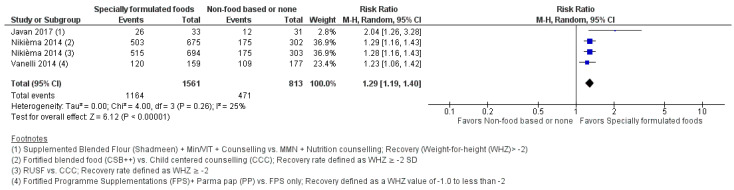
Forest plot of specially formulated foods compared to non-food-based approaches or none. Outcome: anthropometric recovery rate [20,22,23].

Analysis of the four studies included in this comparison category [20,22,23,24] suggests that SFFs, when compared to non-food-based interventions or none, likely increase the recovery rate (RR: 1.29; 95% CI: 1.19 to 1.40; *n* = 2348; three studies; moderate certainty evidence; Figure 4) and have little or no effect on deterioration to severe wasting (RR: 0.78; 95% CI: 0.59 to 1.03; *n* = 1974; one study; moderate certainty evidence). SFFs may increase WHZ (MD: 0.32; 95% CI: 0.18 to 0.45; *n* = 365; two studies; low certainty evidence), WAZ (MD: 0.26; 95% CI: 0.14 to 0.38; *n* = 365; two studies; low certainty evidence), MUAC gain (MD: 0.25 cm; 95% CI: 0.09 to 0.41; *n* = 301; one study; low certainty evidence), and weight gain (MD 0.26 g/kg/day; 95%CI: 0.11 g/kg/day to 0.41 g/kg/day; *n* = 64; low certainty evidence), but may have little or no effect on the height for age z-score (HAZ) (MD: 0.10; 95% CI: 0.00 to 0.19; *n* = 365; two studies; low certainty evidence). SFFs likely reduce non-response by 52% (RR: 0.48; 95% CI: 0.39 to 0.60; *n* = 1974; one study; moderate certainty evidence) and may decrease the time to recovery (MD: −1.12 weeks; 95% CI: −2.10 to −0.14; *n* = 1368; one study). The impact on height gain and mortality was uncertain. The effect on sustained recovery was not reported. Forest plots for all the outcomes are shown in Supporting Figures S2–S12 and the full evidence profile can be found in Supporting Table S3.

Subgroup analysis by a comparison group (i.e., by standard or care group or by counselling) suggests an increase in the recovery rate (RR: 1.29; 95% CI: 1.19 to 1.39; one study) when SFFs were compared with counselling (Supporting Figure S13). However, there was an uncertain effect of specially formulated food on mortality when compared with the standard of care or counselling only (Supporting Figure S14).

Sensitivity analysis was performed for the comparison specially formulated food vs. other or none by excluding studies with unclear risk of bias for both sequence generation and allocation concealment. Notably, the removal of the study by Javan et al., 2017, did not show a significant change in the recovery rate, WHZ, WAZ and HAZ [20].

#### 3.4.2. Comparison 4: Multicomponent Intervention versus Non-Food-Based or None

**Figure 5:**
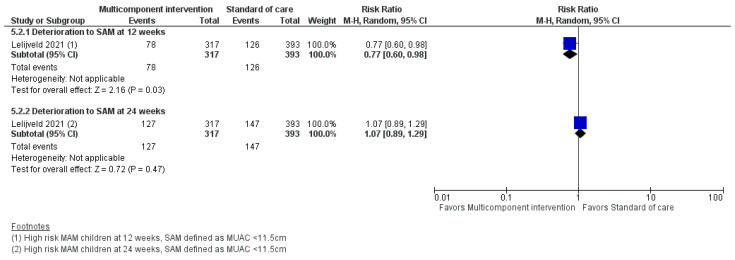
Forest plot of multicomponent intervention compared to non−food−based or none. Outcome: deterioration to SAM [21].

The findings from one study [21] found an uncertain effect of a multicomponent intervention provided to high-risk MAM children only, on recovery rate after 12 and 24 weeks of enrolment when compared to standard of care. The multicomponent intervention may decrease deterioration to severe wasting by 23% (RR 0.77; 95% CI: 0.6 to 0.98; one study; *n* = 710; Figure 5) after 12 weeks of intervention, but it may have little or no effect on deterioration to severe wasting after 24 weeks of enrollment. The multicomponent intervention may also have little or no effect on WHZ, WAZ, HAZ, MUAC gain after 12 and 24 weeks of enrollment. It may increase weight by 0.12 g/kg/day (MD 0.02 g/kg/day; 95% CI: 0.22 to 0.22; one study; *n* = 710) after 24 weeks of enrollment but may have little or no effect on weight gain after 12 weeks of enrollment and on non-response and mortality after 12 and 24 weeks of enrollment. The study did not report on height gain, sustained recovery, and on time to recovery. Forest plots for all the outcomes are shown in Supporting Figures S15–S23 and the full evidence profile can be found in Supporting Table S4.

## 4. Discussion

### 4.1. Summary of Results

This systematic review summarizes findings from five studies, all of which are RCTs. Evidence indicates that providing SFFs, regardless of formulation or dosing, to children with MAM likely increases recovery, decreases non-response with no effect on deterioration to SAM when compared to other approaches (i.e., counselling) or no intervention. There is low certainty evidence that interventions with SFFs may reduce time to recovery and may increase total weight gain, WHZ, WAZ and MUAC. The intervention may not have an effect on height and HAZ gain. The certainty of evidence relating to mortality was judged to be very low, as none of the studies were sufficiently powered to assess it. Death as an outcome tends to be a rare event in MAM treatment programs. Thus, studies would need to include an extremely large sample size in order to achieve sufficient statistical power to include mortality as a primary outcome, which is often impractical.

The evidence of the multicomponent intervention with RUTF and antibiotics compared to counselling in high-risk MAM children only indicates that it may have little or no effect on recovery, WHZ, WAZ, HAZ and non-response after 12 weeks of intervention, that the multicomponent intervention may decrease deterioration to severe wasting after 12 weeks of intervention and likely has little or no effect on MUAC and weight gain. However, this study was not powered for subgroup analysis of high-risk MAM children.

We did not find any study reporting on comparison 3, i.e., specially formulated foods vs. non-specially formulated foods

### 4.2. Overall Completeness and Applicability

The existing body of evidence is quite small given only five studies were identified to fit the inclusion criteria for this analysis. Furthermore, these studies only addressed three of the four different comparisons that we sought to conduct. In these studies, the “other approaches” included nutrition counselling alone or standard of care, which was either nutrition counselling with a vitamin mineral supplement and/or a general food ration for malnourished children and/or growth monitoring. Despite differences in interventions in the control group, results and direction of effect remained consistent. While studies were few and specific to the certain contexts in which they were implemented, results may be applicable to other contexts, since nutrition counselling and general food rations are common alternatives to MAM treatment. It is however important to note that both nutrition counselling and general food rations can vary significantly across programs and contexts, which could influence the results of further comparisons.

No studies were identified to assess the effectiveness of SFFs compared to non-specially formulated foods, nor were any identified regarding a comparison between non-SFFs versus other interventions.

Given the small number of studies, we were not able to carry out subgroup analyses to determine which categories of MAM children require SFFs. Nonetheless, the Lelijveld et al. (2021) study [21], by design, aimed to investigate an intervention based on risk stratification within MAM children. While the study was designed to provide a package of interventions to children deemed “high-risk”, the authors noted that the criteria for identifying risk may not have been completely appropriate as two criteria (“Mother not the primary caregiver” and “less than 2 years and not breastfeeding”) were identified as not being correlated with an increased risk of deterioration. Rather, having a low MUAC for children under 12 months, history of SAM and being a twin were found to be significant risk factors for deterioration among control group children [21]. Two observational studies from Ethiopia not included in this systematic review identified similar risk factors as those noted by the authors in the Lelijveld et al. (2021) study. The first includes a prospective study that followed MAM children without treatment for seven months and found 54% of children recovered, 32.5% remained MAM, and 9.3% encountered a single episode of SAM. Risk factors of poor outcomes, including no recovery from MAM over the seven month period and/or deterioration to SAM, consisted of having a low MUAC and/or low WHZ at baseline, poor child feeding practices, and household food insecurity [25]. A second prospective cohort study in Ethiopia found that without treatment, approximately two-thirds of children from food-insecure and one-third from food-secure households, did not experience recovery from MAM. Dietary diversity and maternal factors, and maternal MUAC demonstrated to be associated with adverse health outcomes [26]. Worse anthropometric measurements upon admission and discharge as well as household food insecurity were cited as factors associated with poor post-discharge outcomes as well in two longitudinal studies investigating relapse in Malawi [27,28]. These studies indicate that moderately wasted children from vulnerable households and those with lower anthropometry status could be at greater risk for poor outcomes and may benefit from more targeted treatment strategies. Other indicators, such as multiple anthropometric deficits, captured by low WAZ, have been shown to be associated with high risk of mortality [29]. A study by Myatt et al. (2019) using data from Senegal found that low WAZ and MUAC were independently associated with near-term mortality [30]. The study also established that a combination of MUAC and WAZ successfully identified all near-term deaths associated with severe anthropometric deficits, encompassing concurrent wasting and stunting [30]. Consistent with these findings, authors of the Lelijveld et al. (2021) study found that a combination of three risk factors: MUAC < 12.0 cm, WAZ < −3, and declining anthropometry during treatment for MAM can effectively predict 90% of cases leading to deterioration into SAM with a specificity rate of 67% in context of Sierra Leone [21]. Further trials are required to confirm the efficacy and cost-effectiveness of prioritizing treatment to children with such risks.

As there were too few studies in this review to allow for subgroup analysis to answer the question of whom should be prioritized for treatment, the WHO commissioned a prognostic factor systematic review as part of the guideline development process to explore the question of who might most benefit from supplementary foods separately [31]. Through this process a series on individual child factors and social factors were identified, namely, which are in line with the above-mentioned studies. These include a MUAC between 115–119 mm, WAZ < −3 SD, age < 24 months, failing to recover from moderate wasting after receiving other interventions (e.g., counselling alone), having relapsed to moderate wasting, history of severe wasting, co-morbidity and personal circumstances such as mother died or poor maternal health and well-being [31].

### 4.3. Agreements and Disagreements with Other Studies or Reviews

The findings of this systematic review are consistent with previously published systematic reviews on the topic. A recent systematic review conducted in 2020 explored effectiveness of food interventions versus no treatment or nutrition counselling in the management of moderate wasting [32]. Of the included studies, seven concluded that food products demonstrated better anthropometric outcomes in comparison to counseling and/or micronutrient supplementation. Conversely, two studies did not identify a significant advantage of food product interventions, and an additional two studies yielded inconclusive results. The inclusion criteria used in this systematic review were less strict than the ones used in the current analysis, therefore additional studies were identified and included, i.e., studies that did not disaggregate by MAM status as well as studies that only had a small proportion of MAM children were included. It is also worth noting that the studies that did not find an effect only included a small proportion of MAM children and did not disaggregate results by MAM status. In line with our findings, the authors concluded that evidence suggests that supplementary foods indeed contribute to recovery and exhibit to be more effective than counselling; however, the specific type and duration of the supplementary food provided are crucial factors to be considered. Furthermore, the authors highlighted the potential to enhance the effectiveness by focusing on improving the quality and adherence to counseling interventions. A more recent systematic review by Gluning et al. (2021) also found that children had a greater likelihood of recovery if they received a specially formulated food than if they did not [33].

### 4.4. Implications for Practice

The findings of this systematic review indicate that SFFs are likely beneficial for children with moderate wasting in terms of recovery and other anthropometric outcomes. While the primary aim of wasting interventions is to save lives, any specific recommendations for the use of SFFs in the treatment of MAM may also want to consider potential impacts on other outcome indicators, such as body composition and functional outcomes. Concerns have been raised regarding a potential impact of routinely providing specially formulated lipid-based products (e.g., RUSF and RUTF) to children with MAM on increasing obesity rates, given the escalating challenge of the double burden of malnutrition, whereby populations are experiencing both high rates of undernutrition and also growing rates of overweight. While this review was limited to outcomes in anthropometry and mortality, to our knowledge there is no evidence supporting concerns that treatment of moderately wasted children would contribute to an increase in population-wide rates of overweight and obesity. The study by Lelijveld et al. (2021) did not find any evidence indicating excessive or unhealthy weight gain (based on skinfold thickness) in individuals, in those who received RUTF compared with those who did not receive it [21]. The mean z-scores for skinfold thickness in both groups remained below the global average [21]. This was similar to findings of a study in Kenya, which found that children undergoing treatment for MAM with RUTF exhibited comparable body composition to those treated with RUSF and neither group revealed excessive adiposity [34]. Furthermore, two RCTs comparing fortified blended foods to lipid-based products for treatment also indicated the absence of any adverse effects on body composition [35,36].

Cost-effectiveness is also a practically relevant outcome of interest given many resource constraints in areas where prevalence of child moderate wasting is high. Unfortunately, information to date on cost and cost-effectiveness of acute malnutrition treatment is limited [37]. Among the studies included in this review, only Lelijveld et al. (2021) mentioned costs, and even so, such data were limited to RUTF costs only. Nonetheless, the study found similar RUTF costs per child treated (USD13 and USD19) and recovered (USD27 and USD23) at the intervention and control sites, respectively. Other studies testing a range of supplements for treatment of MAM found supplements costs ranged from USD5 to USD17 per child [38,39,40], while total program costs ranged from USD28-USD112 per child treated [39,41], where a large variation in costs can be explained by contexts as well as which costs are included in the overall estimate. One study found the treatment of MAM with RUSF to be cost-effective [39].

### 4.5. Implications for Further Research

The available evidence to answer the research questions included in this review is very limited, leaving obvious gaps in the evidence-base that require further research. Specifically, studies are needed that compare non-specially formulated foods/dietary approaches to other approaches (including counselling or cash) and that compare SFFs to non-specially formulated foods. Formulations and dosing of supplements provided in the intervention groups of the four studies differed significantly. Additional research is required to ascertain the optimal type, composition, and dosage of supplements in terms of both effectiveness and cost-effectiveness.

Only one of the studies in this review [23] achieved a recovery rate > 75%, meeting the Sphere minimum standard for recovery in MAM programs [42]. Nikièma et al. found recovery rates of 74.5% and 74.2% in the CSB++ and RUSF arms, respectively. In the intervention groups of the studies by Javan et al. (2017) and Lelijveld et al. (2021), 68% and 48% recovered, respectively [20,21]. In the control groups recovery rates ranged from 31.6% to 61.6% [20,21,22,23]. Further research is needed to determine what programmatic, contextual, or other barriers exist for programs to reach overall higher recovery rates and whether the definition of recovery is appropriate and achievable in all populations.

This review incorporated three studies that included a counseling intervention as part of their research design. In two studies, program attendance and protocol adherence were lower in the counselling arms than in the arms that provided the SFFs [21,22]. In an explorative restricted analysis on non-defaulter children, Nikièma et al. stated that when analyses were adjusted for attendance, the differences between food-supplementation groups and the child-centered counselling groups diminished. Interviews with a subset of women who were less adherent or had dropped out from the program revealed that many did not perceive the advice as useful and encountered difficulty in cooking recommended recipes, given their dependence on their husbands to buy the ingredients. Additionally, the extended waiting time at the healthcare center on the day of counselling was identified as a demotivating factor [22]. Further research is essential to gain a more comprehensive understanding of the impact pathway of counselling and if effectiveness could be improved either through provision of incentives to increase adherence or by better adapting the content or modality of counselling to local causes of malnutrition and dynamics within households. It would also be worth exploring whether a more holistic package of interventions with more focus on morbidity as a cause could improve outcomes of MAM programs. Very high rates of morbidity and inflammation have been found in children with MAM [38,43], but that to date limited attention is placed on the detection and management of illness in children with MAM beyond routine medication which usually includes deworming, vitamin-A, and iron and folic acid supplementation.

Given the large number of children affected by MAM every year, the likely heterogeneity of this population, and the cost of treatment, prioritization of treatment may be needed in some contexts. As mentioned above, this review was not able to conclusively answer the question of whom should be prioritized for treatment. Therefore, a separate prognostic review was commissioned by the WHO, which identified a number of individual and social factors to help determine which MAM children should be prioritized for use of supplementation with specifically formulated foods [31], but noted that research is needed to evaluate the response to interventions in MAM children who have identified prognostic factors and that the prognostic factor review was limited to findings primarily from African contexts [31]. Research should also be conducted to determine the cost-effectiveness of a risk stratification approach in various contexts, which will likely differ across contexts. Cost-effectiveness studies should cost longer term programs, include wider health system costs as well as those for treatment of complicated and uncomplicated SAM, given the impact that treating MAM early can have on the prevention of SAM and duration of treatment.

## 5. Conclusions

Although data are limited, our results suggest that the provision of specially formulated food may be beneficial for children with MAM. We were not able to answer the question of who should be prioritized for treatment as part of this review due to the paucity of data. The findings of this review contributed to the WHO guideline formulation pertaining to the prevention and management of wasting and nutritional oedema in infants and children, which now recommends that in humanitarian crisis or high-risk contexts all children should be considered for a supplementary food in addition to counseling and the provision of home foods. The guideline also highlights a number of individual and social factors that can aid in prioritizing children for treatment, but acknowledged the need for further research.

## Figures and Tables

**Table 1:** Eligibility criteria.

Criteria	Details
**Study Participants**	Children aged over six months with MAM, defined as WHZ ≥ −3 and <−2 and/or a MUAC of ≥11.5 cm and <12.5 cm or a WHZ between >70% and <80% of the median and without any oedema, treated either as inpatients or outpatients.
**Intervention**	Food-based approaches for the treatment of MAM with or without SFFs, including multicomponent interventions.
**Comparison**	Intervention with non-specially formulated foods, non-food based interventions, standard of care or none.
**Outcomes**	Anthropometric recovery, anthropometric outcomes (such as WHZ, weight for age z-score (WAZ), MUAC, weight and height gain), non-response, sustained recovery, recovery time, deterioration to SAM, and mortality.
**Type of Study**	Included: Experimental studies (Randomized controlled trials (RCTs) both individual and cluster randomised and quasi experimental/non-RCTs).Excluded: Observational studies, animal studies, grey literature, reviews, conference abstracts, and studies with external comparison groups.

**Table 2:** Characteristics of the included studies.

Author, Year	Study Design and Setting	Participants, Admission and Recovery Criteria	Description of Intervention and Control Groups	Outcomes
Hossain et al., 2011 [24]	RCT	507 participants of which 301 had a WHZ < −2 and ≥−3. In this review we included data only from the subsample of 301 children who met the wasting criteria. Dhaka Hospital and four community clinics in police Districts of Demra, Gulshan, Sabujbagh, and Mirpur.	Hospital Control (H-C): Fortnightly follow-up care at the International Center for Diarrheal Disease Research, Bangladesh Hospital, including growth monitoring, health education, and micronutrient supplementation. Community control (C-C): Fortnightly follow-up at community clinics, using the same treatment regimen as group H-C. Supplementary food group (C-SF): Community-based follow-up as per group C-C plus cereal-based supplementary food (SF). Psychosocial stimulation: Follow-up as per group C-C plus psychosocial stimulation (PS). Multicomponent intervention (C-SF+PS): Follow-up as per group C-C plus both SF and PS. Duration: 12 weeks.	Weight-for-age (WAZ), weight-for-length (WHZ), height-for-age (HAZ), weight
Nikièma et al., 2014 [22]	cRCT Rural health centres in Hounde, Burkina Faso	1974 children aged 6–24 months with a WHZ < −2 and ≥−3 Recovery was defined as a WHZ ≥ −2 at the end of a 12\week period	Group 1: Child-centered nutrition counselling (CCC) only (*n* = 605). Group 2: 273 kcal/day of corn soy blend ++ (CSB++) (*n* = 675). Group 3: 258 kcal/day of a locally produced peanut and soy-based ready-to-use supplementary food (RUSF) (*n* = 694). Duration: 12 weeks.	Number of children recovered, died, or dropped out, attendance, time to recovery, weight, length, and daily mid upper arm circumference (MUAC) gains
Javan et al., 2017 [20]	Randomised investigator blinded (single blind) controlled trial 17 Health centers in Urban areas of Sabzevar, Iran	70 children aged 9–24 months with MAM defined as WHZ < −2 and ≥−3. Recovery was defined as WHZ > −2 at the end of intervention period	Intervention group: 273 kcal/day of a blended supplementary food consisting of chickpea, barley, wheat rice and sugar. This food was not fortified and was provided in addition to multivitamins/mineral supplement and nutritional counselling. Control group: Multivitamins/mineral supplement and nutritional counselling. Duration: 12 weeks.	Rate of weight gain, length gain and Z-score WHZ gain, recovery proportion and adverse events.
Lelijveld 2021 [21]	cRCT 22 community nutrition clinics in Pujehun District, Sierra Leone	1322 children aged 6–59 months with MAM, defined as MUAC ≥ 11.5 and <12.5 cm without oedema or clinical complications, Only 710 of these were in the High-risk group which met inclusion criteriaRecovery was defined as MUAC > 12.5 cm two consecutive visits	Intervention group: high-eisk MAM children were given 1 sachet of RUTF (92 g, 520 kcal) and a seven-day course of amoxicillin (40–45 mg/kg per dose twice daily) along with standard nutrition counselling. Children with low-risk MAM received standard nutrition counselling only. Control group: 6 weeks of nutrition counselling alone (delivered via biweekly mother support groups led by a respected community elder). Duration: 2–12 weeks.	MUAC, WAZ, HAZ, WHZ, weight gain Kg, Subscapular skinfold thickness for age, triceps skinfold thickness for age, skinfold thickness ratio, recovered, died, deteriorate to SAM, Remained with MAM, recent illness
Vanelli et al., 2014 [23]	RCT Outpatient clinics in Makeni, Northern region, Sierra Leone	332 children aged 6–60 months with a WHZ < −2 and ≥−3. Recovery was defined as achieving a WHZ-score > −2.	Group 1: World Food Programme Feeding Program supplementations (*n* = 177). These consisted of corn flour, palm oil, dried fishes, and milk powder. The ration for one child provided a maximum of 1000 to 1200 kcal/person/day and 10–12% of energy from protein. Group 2: In addition to the above-mentioned food ration, children received a locally produced lipid-based paste called “Parma pap” based on the standard RUTF recipe provided in a dose of 200 kcal/kg/day (*n* = 159). Duration: 12 weeks.	Weight, length, WHZ

## Data Availability

The data presented in this study are available in Supplementary Documents.

## Supplementary Materials

The following supporting information can be downloaded at: https://www.mdpi.com/article/10.3390/nu15173781/s1, Supporting Table S1. Search Strategy; Supporting Table S2. PRISMA Checklist; Supporting Table S3. Evidence profile for comparison 2 (Specially formulated foods compared to non-food-based approaches or none); Supporting Table S4. Evidence profile for comparison 4 (multicomponent intervention versus non-food-based or none); Supporting Figure S1a. Quality of included studies—Cluster randomized trials, Supporting Figure S1b. Quality of included studies—Individually randomized trials; Supporting Figure S2. Forest plot of specially formulated foods compared to non-food-based approaches or none. Outcome: Weight-for-height z-score; Supporting Figure S3. Forest plot of specially formulated foods compared to non-food-based approaches or none. Outcome: Deterioration to SAM; Supporting Figure S4. Forest plot of specially formulated foods compared to non-food-based approaches or none. Outcome: Weight-for-height z-score; Supporting Figure S5. Forest plot of specially formulated foods compared to non-food-based approaches or none. Outcome: Weight-for-age z-score; Supporting Figure S6. Forest plot of specially formulated foods compared to non-food-based approaches or none. Outcome: Height-for-age z-score; Supporting Figure S7. Forest plot of specially formulated foods compared to non-food-based approaches or none. Outcome: MUAC gain; Supporting Figure S8. Forest plot of specially formulated foods compared to non-food-based approaches or none. Outcome: Weight gain; Supporting Figure S9. Forest plot of specially formulated foods compared to non-food-based approaches or none. Outcome: Height gain; Supporting Figure S10. Forest plot of specially formulated foods compared to non-food-based approaches or none. Outcome: Non-response; Supporting Figure S11. Forest plot of specially formulated foods compared to non-food-based approaches or none. Outcome: Time to recovery; Supporting Figure S12. Forest plot of specially formulated foods compared to non-food-based approaches or none. Outcome: Mortality; Supporting Figure S13. Forest plot of specially formulated foods compared to non-food-based approaches or none. Outcome: Recovery rate (subgroup analysis based on type of comparison group); Supporting Figure S14. Forest plot of specially formulated foods compared to non-food-based approaches or none. Outcome: Mortality (subgroup analysis based on type of comparison group); Supporting Figure S15: Forest plot of multicomponent intervention compared to non-food-based or none. Outcome: Recovery rate; Supporting Figure S16: Forest plot of multicomponent intervention compared to non-food-based or none. Outcome: Deterioration to SAM; Supporting Figure S17: Forest plot of multicomponent intervention compared to non-food-based or none. Outcome: Weight for height z-score; Supporting Figure S18: Forest plot of multicomponent intervention compared to non-food-based or none. Outcome: Weight for age z-score; Supporting Figure S19: Forest plot of multicomponent intervention compared to non-food-based or none. Outcome: Height for age z-score; Supporting Figure S20: Forest plot of multicomponent intervention compared to non-food-based or none. Outcome: MUAC gain; Supporting Figure S21: Forest plot of multicomponent intervention compared to non-food-based or none. Outcome: Weight gain; Supporting Figure S22: Forest plot of multicomponent intervention compared to non-food-based or none. Outcome: Non-response; Supporting Figure S23: Forest plot of multicomponent intervention compared to non-food-based or none. Outcome: Mortality.
