# Supplementary material for: Systematic Review of Management of Moderate Wasting in Children over 6 Months of Age
Source: Nutrients. 2023 Aug 30;15(17):3781. doi: 10.3390/nu15173781 (PMC10490450; doi:10.3390/nu15173781)
Supplement: Supplementary file 1 [file nutrients-15-03781-s001.zip › Annex 1. Supporting Tables.pdf]

SUPPORTING TABLES

Supporting Table S1. Search Strategy

Children	AND	Wasting	AND	Management
"Infant" [MeSH] OR "Child, preschool" [MeSH] OR<br><br>Infant* OR toddler* OR baby OR babies OR preschool OR newborn* OR neonate* OR kindergarten OR under-5* OR "under 5*" OR under-five OR "under five" OR kid* OR kids paediatr* OR pediatr* OR child*		"malnutrition" [MeSH] OR "infant nutrition disorers" [MeSH] OR "protein-energy malnutrition" [MeSH] OR "wasting syndrome" [MeSH] OR malnourish* OR undernutrition OR wasting OR "Acute Malnutrition"[MeSH] OR MAM OR undernutrition OR "Protein-Energy Malnutrition"[MeSH]<br><br>OR under-nutrition OR underweight OR wast* OR "weight for height" OR "weight-for-height" OR "weight for length" OR "weight-for-length" OR "weight for age" OR "weight-for-age" OR "mid-upper arm circumference" OR "mid upper arm circumference" OR MUAC		"Food" [MeSH] OR "infant food" [MeSH] OR "food, fortified" [MeSH] OR "food, formulated" [MeSH] OR "dietary supplements" [MeSH] OR "dietary fat*" [MeSH] OR "Milk Proteins" [Mesh] OR "fortified food*" OR diet* OR supplement* OR "ready to use therapeutic food" OR RUTF OR "ready to use supplementary food" OR RUSF OR "ready to use food" OR RUF OR F100 OR F75 OR CTC OR "micronutrient* supplement*" OR fat* OR "dietary fat*" OR "dietary protein*" OR FBF OR "corn soy" OR "Wheat soy* blend*" OR "Rice milk blend*" OR "Milk rice blend*" OR "Pea wheat blend*" OR "Cereal pulse blend*" OR "Lipid-based nutrient supplement*" OR Nutributter OR CSB OR Supercereal* OR (diet* adj3 supplement*) OR "lipid based nutrient supplement*" (supplement* adj3 food*) OR "ready to use" OR ready-to-use OR RUTF OR RUSF OR RUF OR F100 OR F75 OR CTC OR FBF OR CMAM OR "community based management" OR "community-based management" OR "integrated community case management" OR ICCM OR "integrated management of acute malnutrition" OR IMAM OR "inpatient management" OR "in-patient management" OR IMCI OR IMNCI OR "facility based management" OR "facility-based management" OR "supplementary feeding program*" OR SFP OR incentiv* OR Counsel* OR educati* OR empower* OR (women* adj2 group*) OR cash OR agricultur* OR homestead OR "food security" OR WASH OR "water, sanitation, and hygiene"

Supporting Table S2. PRISMA Checklist.

Section and Topic	Item #	Checklist item	Location where item is reported
**TITLE**			
Title	1	Identify the report as a systematic review.	Page 1
**ABSTRACT**			
Abstract	2	See the PRISMA 2020 for Abstracts checklist.	Page 1
**INTRODUCTION**			
Rationale	3	Describe the rationale for the review in the context of existing knowledge.	Page 1-2
Objectives	4	Provide an explicit statement of the objective(s) or question(s) the review addresses.	Page 2
**METHODS**			
Eligibility criteria	5	Specify the inclusion and exclusion criteria for the review and how studies were grouped for the syntheses.	Page 2-3
Information sources	6	Specify all databases, registers, websites, organisations, reference lists and other sources searched or consulted to identify studies. Specify the date when each source was last searched or consulted.	Page 4
Search strategy	7	Present the full search strategies for all databases, registers and websites, including any filters and limits used.	Page 4-5 and Supporting table 1
Selection process	8	Specify the methods used to decide whether a study met the inclusion criteria of the review, including how many reviewers screened each record and each report retrieved, whether they worked independently, and if applicable, details of automation tools used in the process.	Page 4
Data collection process	9	Specify the methods used to collect data from reports, including how many reviewers collected data from each report, whether they worked independently, any processes for obtaining or confirming data from study investigators, and if applicable, details of automation tools used in the process.	Page 4-5
Data items	10a	List and define all outcomes for which data were sought. Specify whether all results that were compatible with each outcome domain in each study were sought (e.g. for all measures, time points, analyses), and if not, the methods used to decide which results to collect.	Table 1
	10b	List and define all other variables for which data were sought (e.g. participant and intervention characteristics, funding sources). Describe any assumptions made about any missing or unclear information.	Page 5
Study risk of bias assessment	11	Specify the methods used to assess risk of bias in the included studies, including details of the tool(s) used, how many reviewers assessed each study and whether they worked independently, and if applicable, details of automation tools used in the process.	Page 4
Effect measures	12	Specify for each outcome the effect measure(s) (e.g. risk ratio, mean difference) used in the synthesis or presentation of results.	Page 5
Synthesis methods	13a	Describe the processes used to decide which studies were eligible for each synthesis (e.g. tabulating the study intervention characteristics and comparing against the planned groups for each synthesis (item #5)).	Page 6 and Figure 3.

Section and Topic	Item #	Checklist item	Location where item is reported
	13b	Describe any methods required to prepare the data for presentation or synthesis, such as handling of missing summary statistics, or data conversions.	Page 4-5
	13c	Describe any methods used to tabulate or visually display results of individual studies and syntheses.	Page 4-5
	13d	Describe any methods used to synthesize results and provide a rationale for the choice(s). If meta-analysis was performed, describe the model(s), method(s) to identify the presence and extent of statistical heterogeneity, and software package(s) used.	Page 4-5
	13e	Describe any methods used to explore possible causes of heterogeneity among study results (e.g. subgroup analysis, meta-regression).	Page 4-5
	13f	Describe any sensitivity analyses conducted to assess robustness of the synthesized results.	Page 4-5
Reporting bias assessment	14	Describe any methods used to assess risk of bias due to missing results in a synthesis (arising from reporting biases).	Page 4
Certainty assessment	15	Describe any methods used to assess certainty (or confidence) in the body of evidence for an outcome.	Page 4-5
**RESULTS**			
Study selection	16a	Describe the results of the search and selection process, from the number of records identified in the search to the number of studies included in the review, ideally using a flow diagram.	Page 6-7 and Figure 2
	16b	Cite studies that might appear to meet the inclusion criteria, but which were excluded, and explain why they were excluded.	Page 7 and Figure 2
Study characteristics	17	Cite each included study and present its characteristics.	Page 8 and Table 2
Risk of bias in studies	18	Present assessments of risk of bias for each included study.	Supporting Figure 1.
Results of individual studies	19	For all outcomes, present, for each study: (a) summary statistics for each group (where appropriate) and (b) an effect estimate and its precision (e.g. confidence/credible interval), ideally using structured tables or plots.	Figures 4-5 and Supporting figures S2-S23.
Results of syntheses	20a	For each synthesis, briefly summarise the characteristics and risk of bias among contributing studies.	GRADE assessments are mentioned for the synthesis and more detail is provided in supporting

Section and Topic	Item #	Checklist item	Location where item is reported
			tables S4-S5
	20b	Present results of all statistical syntheses conducted. If meta-analysis was done, present for each the summary estimate and its precision (e.g. confidence/credible interval) and measures of statistical heterogeneity. If comparing groups, describe the direction of the effect.	Results are presented in the text page 7-10, figures 4-5 and Supporting figures S2-S23.
	20c	Present results of all investigations of possible causes of heterogeneity among study results.	Presented in the text pages 9-10.
	20d	Present results of all sensitivity analyses conducted to assess the robustness of the synthesized results.	Page 10.
Reporting biases	21	Present assessments of risk of bias due to missing results (arising from reporting biases) for each synthesis assessed.	Assessment of publication bias is covered under “Other considerations” in the GRADE tables. Supporting Tables S3 -S4.
Certainty of evidence	22	Present assessments of certainty (or confidence) in the body of evidence for each outcome assessed.	Certainty of evidence is presented in the results text in pages 7-10 and Supporting Tables S3-S4.
**DISCUSSION**			
Discussion	23a	Provide a general interpretation of the results in the context of other evidence.	Pages 11-14
	23b	Discuss any limitations of the evidence included in the review.	Pages 11-14
	23c	Discuss any limitations of the review processes used.	Pages 11-14

Section and Topic	Item #	Checklist item	Location where item is reported
	23d	Discuss implications of the results for practice, policy, and future research.	Pages 13-14
**OTHER INFORMATION**			
Registration and protocol	24a	Provide registration information for the review, including register name and registration number, or state that the review was not registered.	Page 5
	24b	Indicate where the review protocol can be accessed, or state that a protocol was not prepared.	Page 5
	24c	Describe and explain any amendments to information provided at registration or in the protocol.	N/A
Support	25	Describe sources of financial or non-financial support for the review, and the role of the funders or sponsors in the review.	Page 15
Competing interests	26	Declare any competing interests of review authors.	Page 15
Availability of data, code and other materials	27	Report which of the following are publicly available and where they can be found: template data collection forms; data extracted from included studies; data used for all analyses; analytic code; any other materials used in the review.	All data included in this review is published in the relevant papers. The excel data extraction form can be made available upon request.

Supporting Table S3. Evidence profile for comparison 2 (Specially formulated foods compared to non-food-based approaches or none)

Certainty assessment							№ of patients		Effect		Certainty	Importance
№ of studies	Study design	Risk of bias	Inconsistency	Indirectness	Imprecision	Other considerations	specially formulated foods	non-food based approaches/other	Relative (95% CI)	Absolute (95% CI)		
**Anthropometric recovery at 12 weeks**												
3	randomised trials	serious[a]	not serious	not serious	not serious	none	1164/1561 (74.6%)	471/813 (57.9%)	**RR 1.29** (1.19 to 1.40)	**168 more per 1,000** (from 110 more to 232 more)	⊕⊕⊕◯ Moderate	CRITICAL
**Deterioration to severe wasting at 12 weeks**												
1	randomised trials	serious[b]	not serious	not serious	not serious	none	124/1369 (9.1%)	70/605 (11.6%)	**RR 0.78** (0.59 to 1.03)	**25 fewer per 1,000** (from 47 fewer to 3 more)	⊕⊕⊕◯ Moderate	CRITICAL
**WHZ at 12 weeks**												
2	randomised trials	serious[a]	not serious	not serious	serious[c]	none	157	208	-	MD **0.32 higher** (0.18 higher to 0.45 higher)	⊕⊕◯◯ Low	IMPORTANT
**WAZ at 12 weeks**												
2	randomised trials	serious[a]	not serious	not serious	serious[c]	none	157	208	-	MD **0.26 higher** (0.14 higher to 0.38 higher)	⊕⊕◯◯ Low	IMPORTANT

Certainty assessment							№ of patients		Effect		Certainty	Importance
№ of studies	Study design	Risk of bias	Inconsistency	Indirectness	Imprecision	Other considerations	specially formulated foods	non-food based approaches/other	Relative (95% CI)	Absolute (95% CI)		
**HAZ at 12 weeks**												
2	randomised trials	serious[a]	not serious	not serious	serious[d]	none	157	208	-	MD **0.1 higher** (0 to 0.19 higher)	⊕⊕◯◯ Low	IMPORTANT
**MUAC at 12 weeks (cm)**												
1	randomised trials	serious[e]	not serious	not serious	serious[c]	none	124	177	-	MD **0.25 higher** (0.09 higher to 0.41 higher)	⊕⊕◯◯ Low	IMPORTANT
**Average weight gain (g/kg/d) at 12 weeks**												
1	randomised trials	serious[b]	not serious	not serious	serious[f]	none	33	31	-	MD **0.26 higher** (0.11 higher to 0.41 higher)	⊕⊕◯◯ Low	IMPORTANT
**Average height gain (cm) at 12 weeks**												
1	randomised trials	serious[b]	not serious	not serious	serious[g]	none	33	31	-	MD **0.26 higher** (0.24 lower to 0.76 higher)	⊕⊕◯◯ Low	IMPORTANT
**Sustained recovery - not measured**												
-	-	-	-	-	-	-	-	-	-	-	-	IMPORTANT

Certainty assessment							№ of patients		Effect		Certainty	Importance
№ of studies	Study design	Risk of bias	Inconsistency	Indirectness	Imprecision	Other considerations	specially formulated foods	non-food based approaches/other	Relative (95% CI)	Absolute (95% CI)		
**Non-response at 12 weeks**												
1	randomised trials	serious[b]	not serious	not serious	not serious	none	147/1369 (10.7%)	134/605 (22.1%)	**RR 0.48** (0.39 to 0.60)	**115 fewer per 1,000** (from 135 fewer to 89 fewer)	⨁⨁⨁◯ Moderate	IMPORTANT
**Time to recovery (weeks)**												
1	randomised trials	serious[b]	not serious	not serious	serious[h]	none	1018	350	-	MD **1.12 lower** (2.1 lower to 0.14 lower)	⨁⨁◯◯ Low	IMPORTANT
**Mortality**												
2	randomised trials	serious[a]	not serious	not serious	very serious[i]	none	7/1528 (0.5%)	8/782 (1.0%)	**RR 0.46** (0.17 to 1.28)	**6 fewer per 1,000** (from 8 fewer to 3 more)	⨁◯◯◯ Very low	IMPORTANT

CI: confidence interval; **MD:** mean difference; **RR:** risk ratio

Explanations

a. Serious risk of bias: all included studies had some concerns or high risk of bias; b. Serious risk of bias: the only included study had some concerns of bias; c. Serious imprecision: The 95% CIs around the absolute effect does not cross the null threshold and the MD is large but OIS is modestly breached; d. Serious imprecision: The 95% CIs around the absolute effect crosses the null, ranges from no difference to small benefit; e. Serious risk of bias: the only included study had high risk of bias; f. Serious imprecision: The 95% CI around the absolute effect does not cross the null threshold, however effect ranges from trivial to moderate benefit; g. Serious imprecision: The 95% CIs around the absolute effect crosses the null threshold, including both trivial harm and potentially small benefit; h. Serious imprecision: The 95% CIs around the absolute effect does not cross the null threshold, however the absolute effect includes trivial to moderate benefit; i. Very serious imprecision. Downgraded due to small number of events (does not meet OIS criteria) and large CI.

Supporting Table S4. Evidence profile for comparison 4 (multicomponent intervention versus non-food-based or none).

Certainty assessment							№ of patients		Effect		Certainty	Importance
**№ of studies**	**Study design**	**Risk of bias**	**Inconsistency**	**Indirectness**	**Imprecision**	**Other considerations**	**multicomponent interventions (including specially formulated foods)**	**standard of care**	**Relative (95% CI)**	**Absolute (95% CI)**		
**Anthropometric recovery at 12 weeks**												
1	randomised trials	serious[a]	not serious	not serious	serious[b]	None	134/317 (42.3%)	143/393 (36.4%)	**RR 1.16** (0.97 to 1.40)	**58 more per 1,000** (from 11 fewer to 146 more)	⨁⨁◯◯ Low	CRITICAL
**Anthropometric recovery at 24 weeks (sustained recovery)**												
1	randomised trials	serious[a]	not serious	not serious	serious[b]	None	115/317 (36.3%)	134/393 (34.1%)	**RR 1.06** (0.87 to 1.30)	**20 more per 1,000** (from 44 fewer to 102 more)	⨁⨁◯◯ Low	CRITICAL
**.Deterioration to SAM at 12 weeks**												
1	randomised trials	serious[a]	not serious	not serious	serious[c]	None	78/317 (24.6%)	126/317 (39.7%)	**RR 0.77** (0.60 to 0.98)	**91 fewer per 1,000** (from 159 fewer to 8 fewer)	⨁⨁◯◯ Low	CRITICAL
**Deterioration to SAM at 24 weeks**												

Certainty assessment							№ of patients		Effect		Certainty	Importance
№ of studies	Study design	Risk of bias	Inconsistency	Indirectness	Imprecision	Other considerations	multicomponent interventions (including specially formulated foods)	standard of care	Relative (95% CI)	Absolute (95% CI)		
1	randomised trials	serious[a]	not serious	not serious	serious[d]	None	127/317 (40.1%)	147/393 (37.4%)	**RR 1.07** (0.89 to 1.29)	**26 more per 1,000** (from 41 fewer to 108 more)	⊕⊕◯◯ Low	CRITICAL
**WHZ at 12 weeks**												
1	randomised trials	serious[a]	not serious	not serious	serious[e]	None	317	393	-	MD **0.02 higher** (0.12 lower to 0.16 higher)	⊕⊕⊕◯ Moderate	IMPORTANT
**WHZ at 24 weeks**												
1	randomised trials	serious[a]	not serious	not serious	serious[f]	None	317	393	-	MD **0.09 higher** (0.06 lower to 0.24 higher)	⊕⊕◯◯ Low	IMPORTANT
**WAZ at 12 weeks**												
1	randomised trials	serious[a]	not serious	not serious	serious[f]	None	317	393	-	MD **0.1 higher** (0.05 lower to 0.25 higher)	⊕⊕◯◯ Low	IMPORTANT
**WAZ at 24 weeks**												

Certainty assessment							№ of patients		Effect		Certainty	Importance
№ of studies	Study design	Risk of bias	Inconsistency	Indirectness	Imprecision	Other considerations	multicomponent interventions (including specially formulated foods)	standard of care	Relative (95% CI)	Absolute (95% CI)		
.	randomised trials	serious[a]	not serious	not serious	serious[f]	None	317	393	-	MD **0.14 higher** (0.02 lower to 0.3 higher)	⊕⊕◯◯ Low	IMPORTANT
**HAZ at 12 weeks**												
1	randomised trials	serious[a]	not serious	not serious	serious[f]	None	317	393	-	MD **0.16 higher** (0.02 lower to 0.34 higher)	⊕⊕◯◯ Low	IMPORTANT
**HAZ at 24 weeks**												
1	randomised trials	serious[a]	not serious	not serious	serious[f]	None	317	393	-	MD **0.11 higher** (0.07 lower to 0.29 higher)	⊕⊕◯◯ Low	IMPORTANT
**MUAC at 12 weeks (cm)**												
1	randomised trials	serious[a]	not serious	not serious	not serious[e]	None	317	393	-	MD **0.05 higher** (0.07 lower to 0.17 higher)	⊕⊕⊕◯ Moderate	IMPORTANT
**MUAC at 24 weeks (cm)**												

Certainty assessment							№ of patients		Effect		Certainty	Importance
№ of studies	Study design	Risk of bias	Inconsistency	Indirectness	Imprecision	Other considerations	multicomponent interventions (including specially formulated foods)	standard of care	Relative (95% CI)	Absolute (95% CI)		
1	randomised trials	serious[a]	not serious	not serious	not serious[e]	None	317	393	-	MD **0.1 higher** (0.04 lower to 0.24 higher)	⊕⊕⊕◯ Moderate	IMPORTANT
**Average weight gain at 12 weeks (g/kg/day)**												
1	randomised trials	serious[a]	not serious	not serious	not serious[e]	None	317	393	-	MD **0.13 higher** (0.02 lower to 0.28 higher)	⊕⊕⊕◯ Moderate	IMPORTANT
**Average weight gain at 24 weeks (g/kg/day)**												
1	randomised trials	serious[a]	not serious	not serious	not serious[e]	None	317	393	-	MD **0.12 higher** (0.02 higher to 0.22 higher)	⊕⊕⊕◯ Moderate	IMPORTANT
**Height gain - not measured**												
-	-	-	-	-	-	-	-	-	-	-	-	
**Non-response at 12 weeks**												
1	randomised trials	serious[a]	not serious	not serious	serious[d]	None	89/317 (28.1%)	86/393 (21.9%)	**RR 1.28** (0.99 to 1.66)	**61 more per 1,000** (from 2 fewer to 144 more)	⊕⊕◯◯ Low	IMPORTANT

Certainty assessment							№ of patients		Effect		Certainty	Importance
№ of studies	Study design	Risk of bias	Inconsistency	Indirectness	Imprecision	Other considerations	multicomponent interventions (including specially formulated foods)	standard of care	Relative (95% CI)	Absolute (95% CI)		
**Non-response at 24 weeks**												
1	randomised trials	serious[a]	not serious	not serious	serious[f]	None	32/317 (10.1%)	30/393 (7.6%)	**RR 1.32** (0.82 to 2.13)	**24 more per 1,000** (from 14 fewer to 86 more)	⨁⨁◯◯ Low	IMPORTANT
**Time to recovery - not measured**												
-	-	-	-	-	-	-	-	-	-	-	-	IMPORTANT
**Mortality at 12 weeks**												
1	randomised trials	serious[a]	not serious	not serious	very serious[g]	None	4/317 (1.3%)	12/393 (3.1%)	**RR 0.41** (0.13 to 1.27)	**18 fewer per 1,000** (from 27 fewer to 8 more)	⨁◯◯◯ Very low	IMPORTANT
**Mortality at 24 weeks**												
1	randomised trials	serious[a]	not serious	not serious	Very serious[g]	None	9/317 (2.8%)	20/393 (5.1%)	**RR 0.56** (0.26 to 1.21)	**22 fewer per 1,000** (from 38 fewer to 11 more)	⨁◯◯◯ Very low	IMPORTANT

CI: confidence interval; **MD:** mean difference; **RR:** risk ratio

Explanations

a. Serious risk of bias: All information is from a subsample from only one study with some risk of bias; b. Serious imprecision: The 95% CIs around the absolute effect crosses the null threshold, ranges from important; appreciable benefit to trivial harm; c. Serious imprecision: The 95% CI for the absolute effects does not cross the null threshold but includes a trivial to moderate benefit; d. Serious imprecision: The 95% CI for the absolute effect crosses the null and includes a trivial benefit to moderate harm; e. No serious imprecision: Effect ranging from trivial harm to trivial benefit; f. Serious imprecision: The 95% CI around the absolute effect crosses the null threshold and effect ranges from trivial benefit to small harm; g. Very serious imprecision. Downgraded due to small number of events (does not meet OIS criteria) and large CI.
