# Supplementary material for: Systematic Review of Management of Moderate Wasting in Children over 6 Months of Age
Source: Nutrients. 2023 Aug 30;15(17):3781. doi: 10.3390/nu15173781 (PMC10490450; doi:10.3390/nu15173781)

Supporting information

Supporting Figure S1a. Quality of included studies- Cluster randomised trials.

Study ID	D1a: Randomisation process	D1b: Timing of identification or recruitment of participants	D2: Deviations from the intended interventions	D3: Missing outcome data	D4: Measurement of the outcome	D5: Selection of the reported result	Overall
Lelijveld 2021	+	+	!	+	!	+	!
Nikiema 2014	+	+	+	+	-	+	!

Supporting Figure S1b. Quality of included studies- Individually randomised trials.

Study ID	D1: Randomisation process	D2: Deviations from the intended interventions	D3: Missing outcome data	D4: Measurement of the outcome	D5: Selection of the reported result	Overall
Vanelli 2014	!	+	+	-	-	-
Hossain et al, 2011	+	!	+	!	+	!
Javan et al, 2017	!	!	+	+	+	!

+	Low risk
!	Some concerns
-	High risk

Supporting Figure S2. Forest plot of specially formulated foods compared to non-food-based approaches or none- Outcome: Weight-for-height z-score.

	Specially formulated foods			Non-food based or none				Mean Difference
Study or Subgroup	Mean	SD	Total	Mean	SD	Total	Weight	IV, Random, 95% CI
Hossain 2011 (1)	0.99	0.67	24	0.73	0.76	33	13.1%	0.26 [-0.11, 0.63]
Hossain 2011 (2)	1.25	0.93	18	0.89	0.59	27	7.8%	0.36 [-0.12, 0.84]
Hossain 2011 (3)	0.99	0.67	24	0.69	0.72	29	13.0%	0.30 [-0.07, 0.67]
Hossain 2011 (4)	1.25	0.93	18	0.73	0.76	32	7.2%	0.52 [0.02, 1.02]
Hossain 2011 (5)	0.99	0.67	23	0.89	0.59	27	14.6%	0.10 [-0.25, 0.45]
Hossain 2011 (6)	1.25	0.93	17	0.69	0.72	29	6.9%	0.56 [0.05, 1.07]
Javan 2017 (7)	0.36	0.36	33	0.02	0.52	31	37.5%	0.34 [0.12, 0.56]
**Total (95% CI)**			**157**			**208**	**100.0%**	**0.32 [0.18, 0.45]**

Heterogeneity: $Tau^2 = 0.00$; $Chi^2 = 3.10$, df = 6 ($P = 0.80$); $I^2 = 0\%$
Test for overall effect: $Z = 4.63$ ($P < 0.00001$)

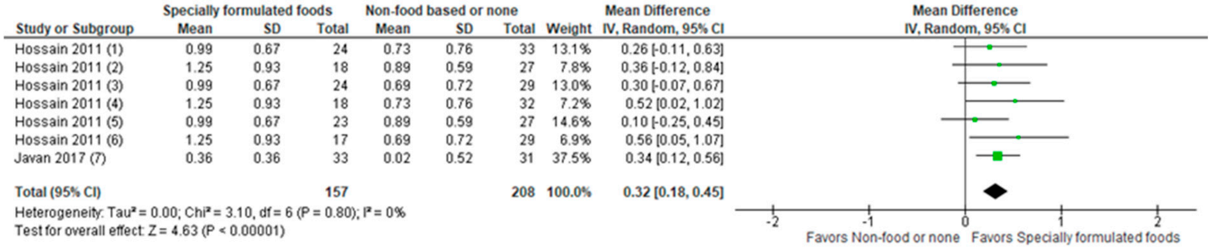

Footnotes
(1) Follow-up at community clinics + cereal-based supplementary food (C-SF) vs. fortnightly follow-up at community clinics (C-C); change in WHZ over a 3 months intervention period
(2) Follow-up at community clinics+ cereal-based supplementary food and psychosocial stimulation (C-SF+PS) vs. C-C plus psychosocial stimulation (C-PS); change in WHZ over 3 months
(3) C-SF vs.followup at health clinics (H-C); change in WHZ over 3 months
(4) C-SF+PS vs C-C; change in WHZ over 3 months
(5) C-SF vs C-PS, Change in WHZ over 3 months
(6) C-SF+PS vs H-C; change in WHZ over 3 months
(7) Supplemented Blended Flour (Shadmeen) + Min/VIT + Counselling vs. MMN + Nutrition counselling; Increase in WHZ from baseline to endline

Supporting Figure S3. Forest plot of specially formulated foods compared to non-food-based approaches or none- Outcome: Deterioration to SAM.

	Specially formulated foods		Non-food based or none			Risk Ratio
Study or Subgroup	Events	Total	Events	Total	Weight	M-H, Random, 95% CI
Nikièma 2014 (1)	67	675	35	302	51.7%	0.86 [0.58, 1.26]
Nikièma 2014 (2)	57	694	35	303	48.3%	0.71 [0.48, 1.06]
**Total (95% CI)**		**1369**		**605**	**100.0%**	**0.78 [0.59, 1.03]**
Total events	124		70			

Heterogeneity: Tau² = 0.00; Chi² = 0.43, df = 1 (P = 0.51); I² = 0%
Test for overall effect: Z = 1.73 (P = 0.08)

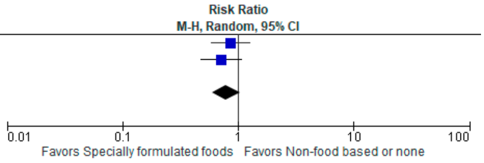

Footnotes
(1) Fortified blended food (CSB++) vs. Child centered counselling (CCC); developed SAM defined as WHZ<-3
(2) RUSF vs. CCC; developed SAM defined as WHZ <-3

Supporting Figure S4. Forest plot of specially formulated foods compared to non-food-based approaches or none- Outcome: Weight-for-height z-score**.**

	Specially formulated foods			Non-food based or none				Mean Difference
Study or Subgroup	Mean	SD	Total	Mean	SD	Total	Weight	IV, Random, 95% CI
Hossain 2011 (1)	0.99	0.67	24	0.73	0.76	33	13.1%	0.26 [-0.11, 0.63]
Hossain 2011 (2)	1.25	0.93	18	0.89	0.59	27	7.8%	0.36 [-0.12, 0.84]
Hossain 2011 (3)	0.99	0.67	24	0.69	0.72	29	13.0%	0.30 [-0.07, 0.67]
Hossain 2011 (4)	1.25	0.93	18	0.73	0.76	32	7.2%	0.52 [0.02, 1.02]
Hossain 2011 (5)	0.99	0.67	23	0.89	0.59	27	14.6%	0.10 [-0.25, 0.45]
Hossain 2011 (6)	1.25	0.93	17	0.69	0.72	29	6.9%	0.56 [0.05, 1.07]
Javan 2017 (7)	0.36	0.36	33	0.02	0.52	31	37.5%	0.34 [0.12, 0.56]
**Total (95% CI)**			**157**			**208**	**100.0%**	**0.32 [0.18, 0.45]**

Heterogeneity: Tau² = 0.00; Chi² = 3.10, df = 6 (P = 0.80); I² = 0%
Test for overall effect: Z = 4.63 (P < 0.00001)

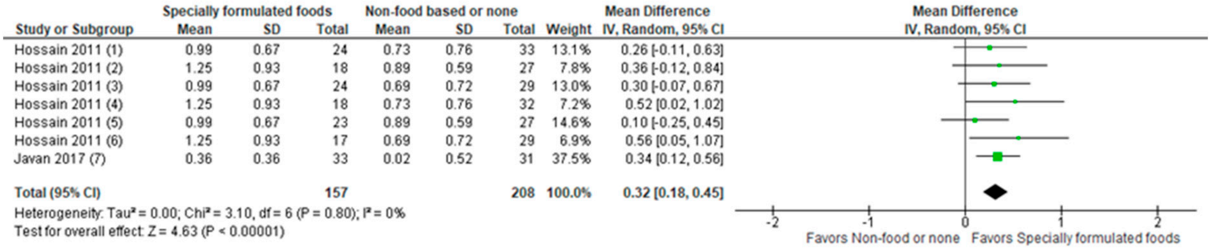

Footnotes
(1) Follow-up at community clinics + cereal-based supplementary food (C-SF) vs. fortnightly follow-up at community clinics (C-C); change in WHZ over a 3 months intervention period
(2) Follow-up at community clinics+ cereal-based supplementary food and psychosocial stimulation (C-SF+PS) vs. C-C plus psychosocial stimulation (C-PS); change in WHZ over 3 months
(3) C-SF vs.followup at health clinics (H-C); change in WHZ over 3 months
(4) C-SF+PS vs C-C; change in WHZ over 3 months
(5) C-SF vs C-PS, Change in WHZ over 3 months
(6) C-SF+PS vs H-C; change in WHZ over 3 months
(7) Supplemented Blended Flour (Shadmeen) + Min/VIT + Counselling vs. MMN + Nutrition counselling; Increase in WHZ from baseline to endline

Supporting Figure S5. Forest plot of specially formulated foods compared to non-food-based approaches or none- Outcome: Weight-for-age z-score.

	Specially formulated foods			Non-food based or none				Mean Difference
Study or Subgroup	Mean	SD	Total	Mean	SD	Total	Weight	IV, Random, 95% CI
Hossain 2011 (1)	1.1	0.53	18	0.84	0.59	33	14.0%	0.26 [-0.06, 0.58]
Hossain 2011 (2)	1.08	0.53	18	1.01	1.12	27	5.9%	0.07 [-0.42, 0.56]
Hossain 2011 (3)	1.1	0.53	17	0.66	0.64	29	11.9%	0.44 [0.10, 0.78]
Hossain 2011 (4)	0.91	0.88	24	0.66	0.64	29	7.9%	0.25 [-0.17, 0.67]
Hossain 2011 (5)	0.91	0.88	24	0.84	0.59	32	8.5%	0.07 [-0.34, 0.48]
Hossain 2011 (6)	0.91	0.88	23	1.01	1.12	27	4.6%	-0.10 [-0.65, 0.45]
Javan 2017 (7)	0.4	0.33	33	0.09	0.37	31	47.3%	0.31 [0.14, 0.48]
**Total (95% CI)**			**157**			**208**	**100.0%**	**0.26 [0.14, 0.38]**

Heterogeneity: Tau² = 0.00; Chi² = 4.42, df = 6 (P = 0.62); I² = 0%
Test for overall effect: Z = 4.31 (P < 0.0001)

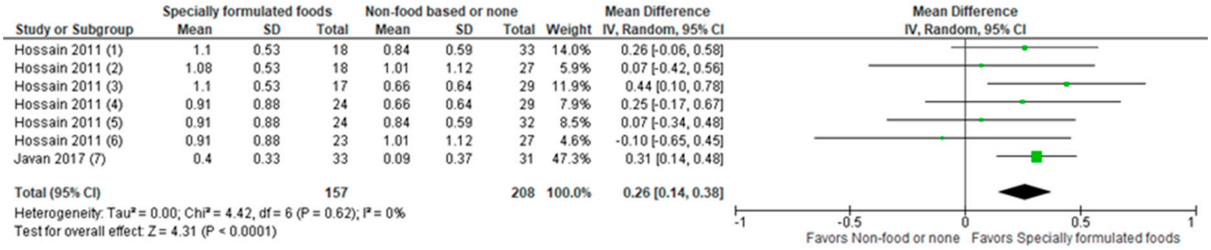

Footnotes
(1) Fortnightly follow-up at community clinics (C-C) plus cereal-based supplementary food plus psychosocial stimulation (C-SF+PS) vs. C-C; Change in WAZ over 3 months
(2) C-SF+PS vs. C-C plus psychosocial stimulation (C-PS); Change in WAZ over 3 months
(3) C-SF+PS vs. fortnightly follow-up at hospital (H-C); Change in WAZ
(4) Follow-up at community clinics + cereal-based supplementary food (C-SF) vs. H-C; Change in WAZ over 3 months
(5) C-SF vs. C-C; Change in WAZ
(6) C-SF vs. C-PS, Change in WAZ over 3 months
(7) Supplemented Blended Flour (Shadmeen) + Min/VIT + Counselling vs. MMN + Nutrition counselling; Change in WAZ over intervention period

Supporting Figure S6. Forest plot of specially formulated foods compared to non-food-based approaches or none- Outcome: Height-for-age z-score.

	Specially formulated foods			Non-food based or none				Mean Difference
Study or Subgroup	Mean	SD	Total	Mean	SD	Total	Weight	IV, Random, 95% CI
Hossain 2011 (1)	-0.21	0.52	23	-0.4	0.36	29	14.6%	0.19 [-0.06, 0.44]
Hossain 2011 (2)	-0.2	0.49	18	-0.4	0.36	29	13.3%	0.20 [-0.06, 0.46]
Hossain 2011 (3)	-0.2	0.49	18	-0.27	0.48	33	11.7%	0.07 [-0.21, 0.35]
Hossain 2011 (4)	-0.2	0.49	17	-0.25	0.48	27	10.5%	0.05 [-0.25, 0.35]
Hossain 2011 (5)	-0.21	0.52	24	-0.25	0.48	27	12.0%	0.04 [-0.24, 0.32]
Hossain 2011 (6)	-0.21	0.52	24	-0.27	0.48	32	12.9%	0.06 [-0.21, 0.33]
Javan 2017 (7)	0.17	0.39	33	0.1	0.39	31	25.0%	0.07 [-0.12, 0.26]
**Total (95% CI)**			**157**			**208**	**100.0%**	**0.10 [0.00, 0.19]**

Heterogeneity: $Tau^2 = 0.00$; $Chi^2 = 1.58$, df = 6 (P = 0.95); $I^2 = 0\%$
Test for overall effect: Z = 2.01 (P = 0.04)

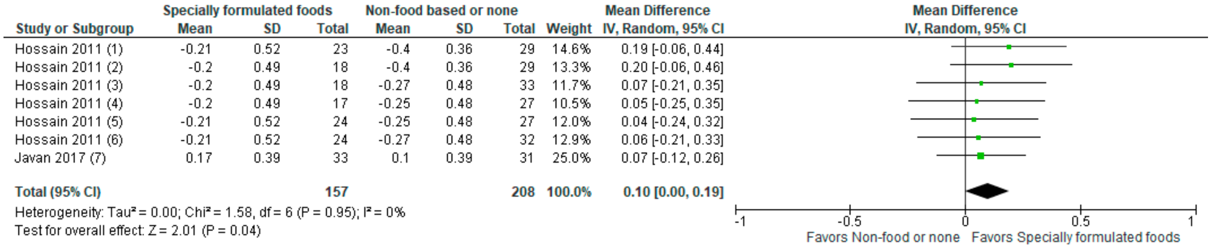

Footnotes
(1) C-SF+PS vs. H-C; Change in HAZ over 3 months
(2) Follow-up at community clinics + cereal-based supplementary food (C-SF) vs.followup at health clinics (H-C)
(3) C-SF vs. fortnightly follow-up at community clinics (C-C); Change in HAZ over 3 months
(4) C-SF vs. C-C plus psychosocial stimulation (C-PS); Change in HAZ over 3 months
(5) Follow-up at community clinics+ cereal-based supplementary food and psychosocial stimulation (C-SF+PS) vs. C-PS; Change in HAZ over the 3 months intervention period
(6) C-SF+PS vs. C-C; Change in HAZ over 3 months
(7) Supplemented Blended Flour (Shadmeen) + Min/VIT + Counselling vs. MMN + Nutrition counselling; Change in HAZ over the intervention period

Supporting Figure S7. Forest plot of specially formulated foods compared to non-food-based approaches or none- Outcome: MUAC gain.

	Specially formulated foods			Non-food based or none				Mean Difference
Study or Subgroup	Mean	SD	Total	Mean	SD	Total	Weight	IV, Random, 95% CI
Hossain 2011 (1)	1.2	0.61	18	1.07	1.07	27	10.5%	0.13 [-0.36, 0.62]
Hossain 2011 (2)	1.06	0.76	24	0.76	0.61	29	18.0%	0.30 [-0.08, 0.68]
Hossain 2011 (3)	1.06	0.76	24	1.07	1.07	27	10.0%	-0.01 [-0.52, 0.50]
Hossain 2011 (4)	1.2	0.61	18	0.9	0.53	33	22.8%	0.30 [-0.03, 0.63]
Hossain 2011 (5)	1.06	0.76	23	0.9	0.53	32	19.6%	0.16 [-0.20, 0.52]
Hossain 2011 (6)	1.2	0.61	17	0.76	0.61	29	19.1%	0.44 [0.07, 0.81]
**Total (95% CI)**			**124**			**177**	**100.0%**	**0.25 [0.09, 0.41]**

Heterogeneity: $Tau^2 = 0.00$; $Chi^2 = 2.68$, df = 5 (P = 0.75); $I^2 = 0\%$
Test for overall effect: Z = 3.07 (P = 0.002)

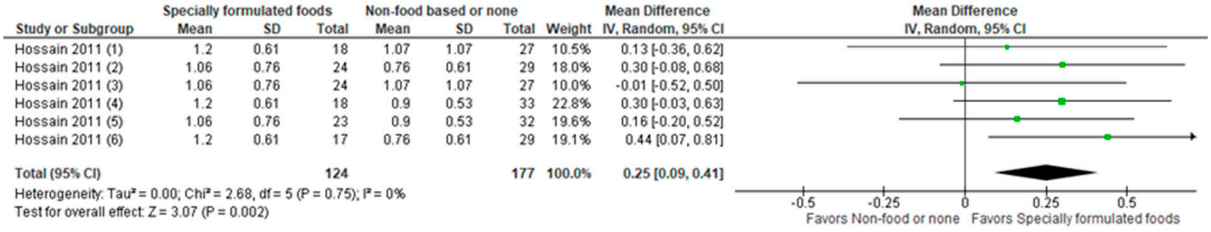

Footnotes
(1) Follow-up at community clinics + cereal-based supplementary food and psychosocial stimulation (C-SF+PS) vs. C-C plus psychosocial stimulation (C-PS); Change in MUAC (cm) over the...
(2) Follow-up at community clinics + cereal-based supplementaryfood (C-SF) vs.followup at health clinics (H-C)
(3) C-SF vs C-PS; Change in MUAC (cm) over the intervention period
(4) C-SF+PS vs. fortnightly follow-up at community clinics (C-C); Change in MUAC (cm) over the intervention period
(5) C-SF vs. C-C; Change in MUAC (cm) over the intervention period
(6) C-SF+PS vs. H-C; Change in MUAC (cm) over the intervention period

Supporting Figure S8. Forest plot of specially formulated foods compared to non-food-based approaches or none- Outcome: Weight gain.

	Specially formulated foods			Non-food based or none				Mean Difference
Study or Subgroup	Mean	SD	Total	Mean	SD	Total	Weight	IV, Random, 95% CI
Javan 2017 (1)	0.81	0.29	33	0.55	0.33	31	100.0%	0.26 [0.11, 0.41]
**Total (95% CI)**			**33**			**31**	**100.0%**	**0.26 [0.11, 0.41]**

Heterogeneity: Not applicable
Test for overall effect: Z = 3.34 (P = 0.0008)

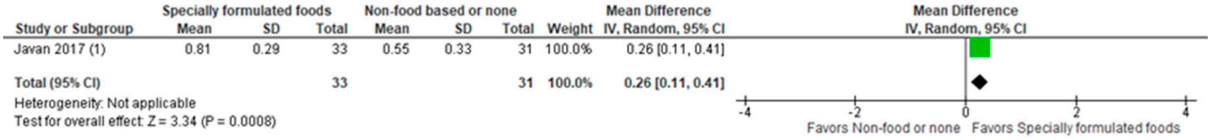

Footnotes
(1) Supplemented Blended Flour (Shadmeen) + Multivitamin and mineral supplement (MMS) + Counselling vs. MMS + Nutrition counselling; Weight gain in grams from baseline to endline

Supporting Figure S9. Forest plot of specially formulated foods compared to non-food-based approaches or none- Outcome: Height gain.

Study or Subgroup	Specially formulated foods Mean	SD	Total	Non-food based or none Mean	SD	Total	Weight	Mean Difference IV, Random, 95% CI
Javan 2017 (1)	3.36	1.04	33	3.1	1.02	31	100.0%	0.26 [-0.24, 0.76]
**Total (95% CI)**			**33**			**31**	**100.0%**	**0.26 [-0.24, 0.76]**
Heterogeneity: Not applicable								
Test for overall effect: Z = 1.01 (P = 0.31)								

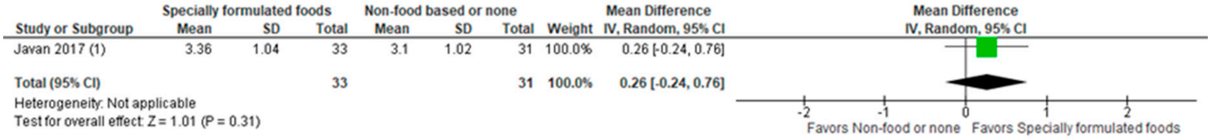

Footnotes
(1) Supplemented Blended Flour (Shadameen) + Multimicronutrient and mineral supplement (MMS) + Counselling vs. MMN + Nutrition counselling; Increment in cm from baseline to endline

Supporting Figure S10. Forest plot of specially formulated foods compared to non-food-based approaches or none- Outcome: Non-response.

Study or Subgroup	Specially formulated foods Events	Total	Non-food based or none Events	Total	Weight	Risk Ratio M-H, Random, 95% CI
Nikièma 2014 (1)	72	694	67	302	49.4%	0.47 [0.35, 0.63]
Nikièma 2014 (2)	75	675	67	303	50.6%	0.50 [0.37, 0.68]
**Total (95% CI)**		**1369**		**605**	**100.0%**	**0.48 [0.39, 0.60]**
Total events	147		134			
Heterogeneity: Tau² = 0.00; Chi² = 0.11, df = 1 (P = 0.74); I² = 0%						
Test for overall effect: Z = 6.64 (P < 0.00001)						

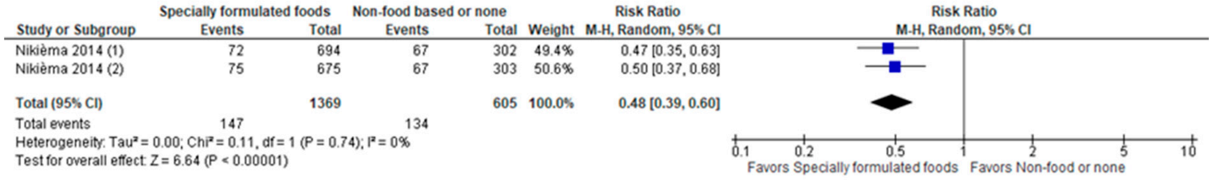

Footnotes
(1) RUSF vs. Child centered counselling (CCC); remained MAM at the end of 3 months intervention
(2) Fortified blended food (CSB++) vs. CCC; remained MAM at the end of 3 months intervention

Supporting Figure S11. Forest plot of specially formulated foods compared to non-food-based approaches or none- Outcome: Time to recovery.

Study or Subgroup	Specially formulated foods Mean	SD	Total	Non-food based or none Mean	SD	Total	Weight	Mean Difference IV, Random, 95% CI
Nikièma 2014 (1)	4.2	5.7	503	5.8	7.4	175	51.8%	-1.60 [-2.80, -0.40]
Nikièma 2014 (2)	5.2	7.2	515	5.8	7.4	175	48.2%	-0.60 [-1.86, 0.66]
**Total (95% CI)**			**1018**			**350**	**100.0%**	**-1.12 [-2.10, -0.14]**
Heterogeneity: Tau² = 0.10; Chi² = 1.26, df = 1 (P = 0.26); I² = 21%								
Test for overall effect: Z = 2.24 (P = 0.03)								

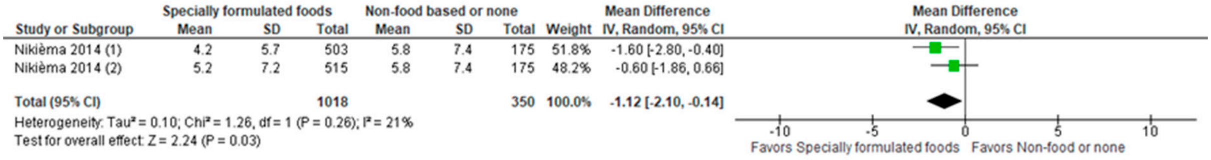

Footnotes
(1) Fortified blended food (CSB++) vs. Child centered counselling (CCC); Time to recovery in weeks
(2) RUSF vs. CCC; Time to recovery in weeks

Supporting Figure S12. Forest plot of specially formulated foods compared to non-food-based approaches or none- Outcome: Mortality.

Study or Subgroup	Specially formulated foods Events	Total	Non-food based or none Events	Total	Weight	Risk Ratio M-H, Random, 95% CI
Nikièma 2014 (1)	3	675	3	302	40.9%	0.45 [0.09, 2.20]
Nikièma 2014 (2)	3	694	3	303	40.9%	0.44 [0.09, 2.15]
Vanelli 2014 (3)	1	159	2	177	18.2%	0.56 [0.05, 6.08]
**Total (95% CI)**		**1528**		**782**	**100.0%**	**0.46 [0.17, 1.28]**
Total events	7		8			
Heterogeneity: Tau² = 0.00; Chi² = 0.03, df = 2 (P = 0.99); I² = 0%						
Test for overall effect: Z = 1.49 (P = 0.14)						

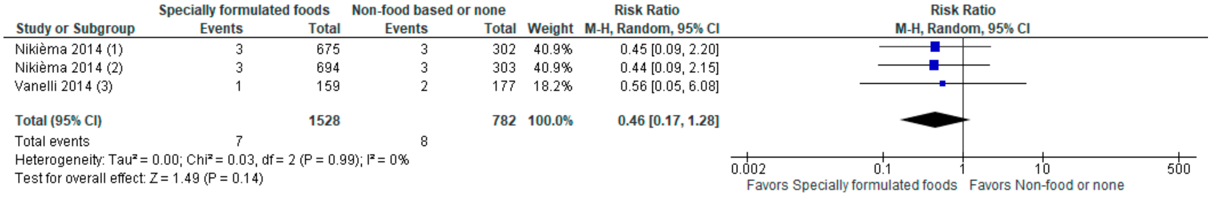

Footnotes
(1) Fortified blended food (CSB++) vs. Child centered counselling (CCC)
(2) RUSF vs. CCC
(3) Food Programme Supplementations (FPS)+ Parma pap (PP) vs. FPS only

Supporting Figure S13. Forest plot of specially formulated foods compared to non-food-based approaches or none- Outcome: Recovery rate (subgroup analysis based on type of comparison group).

	Specially formulated foods		Standard of care/Counselling			Risk Ratio
**Study or Subgroup**	**Events**	**Total**	**Events**	**Total**	**Weight**	**M-H, Random, 95% CI**
**2.1.1 Specially formulated foods vs. Standard of care**						
Javan 2017 (1)	26	33	12	31	2.8%	2.04 [1.26, 3.28]
Vanelli 2014 (2)	120	159	109	177	23.4%	1.23 [1.06, 1.42]
**Subtotal (95% CI)**		**192**		**208**	**26.2%**	**1.50 [0.92, 2.46]**
Total events	146		121			
Heterogeneity: Tau² = 0.10; Chi² = 4.09, df = 1 (P = 0.04); I² = 76%						
Test for overall effect: Z = 1.61 (P = 0.11)						
**2.1.2 Specially formulated foods vs. Counselling**						
Nikièma 2014 (3)	503	675	175	302	36.9%	1.29 [1.16, 1.43]
Nikièma 2014 (4)	515	694	175	303	36.9%	1.28 [1.16, 1.43]
**Subtotal (95% CI)**		**1369**		**605**	**73.8%**	**1.29 [1.19, 1.39]**
Total events	1018		350			
Heterogeneity: Tau² = 0.00; Chi² = 0.00, df = 1 (P = 0.99); I² = 0%						
Test for overall effect: Z = 6.58 (P < 0.00001)						
**Total (95% CI)**		**1561**		**813**	**100.0%**	**1.29 [1.19, 1.40]**
Total events	1164		471			
Heterogeneity: Tau² = 0.00; Chi² = 4.00, df = 3 (P = 0.26); I² = 25%						
Test for overall effect: Z = 6.12 (P < 0.00001)						
Test for subgroup differences: Chi² = 0.37, df = 1 (P = 0.54), I² = 0%						

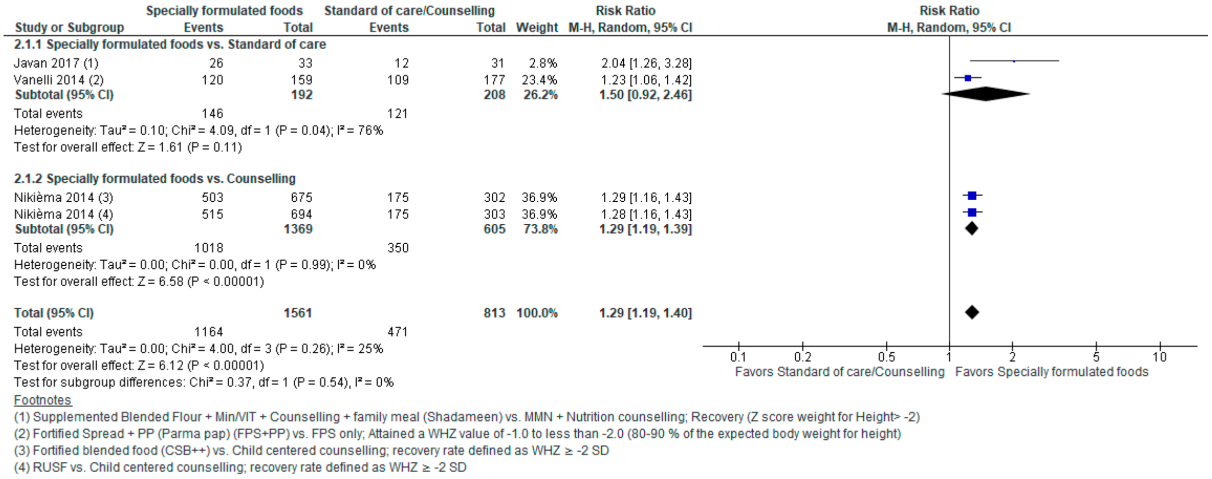

Footnotes
(1) Supplemented Blended Flour + Min/VIT + Counselling + family meal (Shadameen) vs. MMN + Nutrition counselling; Recovery (Z score weight for Height> -2)
(2) Fortified Spread + PP (Parma pap) (FPS+PP) vs. FPS only; Attained a WHZ value of -1.0 to less than -2.0 (80-90 % of the expected body weight for height)
(3) Fortified blended food (CSB++) vs. Child centered counselling; recovery rate defined as WHZ ≥ -2 SD
(4) RUSF vs. Child centered counselling; recovery rate defined as WHZ ≥ -2 SD

Supporting Figure S14. Forest plot of specially formulated foods compared to non-food-based approaches or none- Outcome: Mortality (subgroup analysis based on type of comparison group).

	Specially formulated foods		Standard of care/Counselling			Risk Ratio
**Study or Subgroup**	**Events**	**Total**	**Events**	**Total**	**Weight**	**M-H, Random, 95% CI**
**2.2.1 Specially formulated foods vs. Standard of care**						
Vanelli 2014 (1)	1	159	2	177	18.2%	0.56 [0.05, 6.08]
**Subtotal (95% CI)**		**159**		**177**	**18.2%**	**0.56 [0.05, 6.08]**
Total events	1		2			
Heterogeneity: Not applicable						
Test for overall effect: Z = 0.48 (P = 0.63)						
**2.2.2 Specially formulated foods vs. Counselling**						
Nikièma 2014 (2)	3	694	3	303	40.9%	0.44 [0.09, 2.15]
Nikièma 2014 (3)	3	675	3	302	40.9%	0.45 [0.09, 2.20]
**Subtotal (95% CI)**		**1369**		**605**	**81.8%**	**0.44 [0.14, 1.36]**
Total events	6		6			
Heterogeneity: Tau² = 0.00; Chi² = 0.00, df = 1 (P = 0.98); I² = 0%						
Test for overall effect: Z = 1.42 (P = 0.16)						
**Total (95% CI)**		**1528**		**782**	**100.0%**	**0.46 [0.17, 1.28]**
Total events	7		8			
Heterogeneity: Tau² = 0.00; Chi² = 0.03, df = 2 (P = 0.99); I² = 0%						
Test for overall effect: Z = 1.49 (P = 0.14)						
Test for subgroup differences: Chi² = 0.03, df = 1 (P = 0.86), I² = 0%						

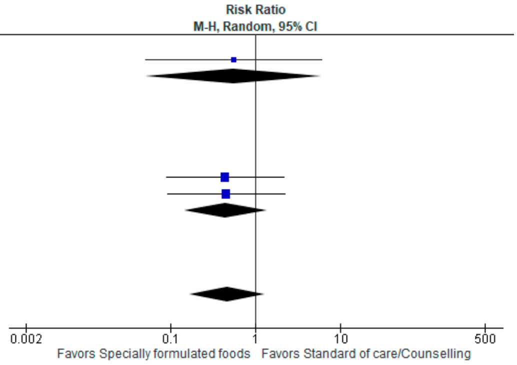

Footnotes
(1) Fortified Spread + PP (Parma pap) (FPS+PP) vs. FPS only
(2) RUSF vs. Child centered counselling
(3) Fortified blended food (CSB++) vs. Child centered counselling

Supporting Figure S15: Forest plot of multicomponent intervention compared to non-food-based or none - Outcome: Recovery rate.

Study or Subgroup	Multicomponent intervention Events	Total	Standard of care Events	Total	Weight	Risk Ratio M-H, Random, 95% CI
**1.1.1 Recovery rate at 12 weeks**						
Lelijveld 2021 (1)	134	317	143	393	100.0%	1.16 [0.97, 1.40]
**Subtotal (95% CI)**		**317**		**393**	**100.0%**	**1.16 [0.97, 1.40]**
Total events	134		143			
Heterogeneity: Not applicable						
Test for overall effect: Z = 1.60 (P = 0.11)						
**1.1.2 Recovery rate at 24 weeks**						
Lelijveld 2021 (2)	115	317	134	393	100.0%	1.06 [0.87, 1.30]
**Subtotal (95% CI)**		**317**		**393**	**100.0%**	**1.06 [0.87, 1.30]**
Total events	115		134			
Heterogeneity: Not applicable						
Test for overall effect: Z = 0.61 (P = 0.54)						

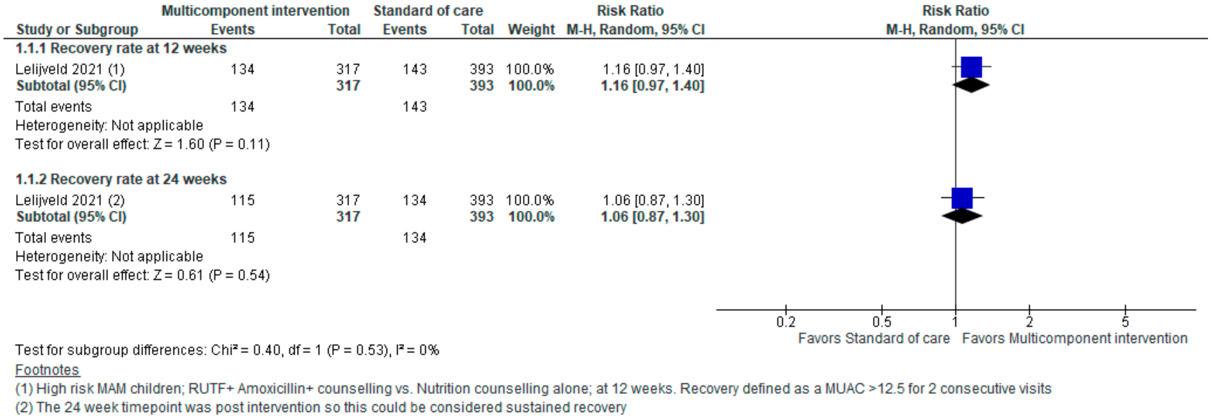

Test for subgroup differences: Chi² = 0.40, df = 1 (P = 0.53), I² = 0%

Footnotes

(1) High risk MAM children; RUTF+ Amoxicillin+ counselling vs. Nutrition counselling alone; at 12 weeks. Recovery defined as a MUAC >12.5 for 2 consecutive visits

(2) The 24 week timepoint was post intervention so this could be considered sustained recovery

Supporting Figure S16: Forest plot of multicomponent intervention compared to non-food-based or none - Outcome: Deterioration to SAM.

Study or Subgroup	Multicomponent intervention Events	Total	Standard of care Events	Total	Weight	Risk Ratio M-H, Random, 95% CI
**1.2.1 Deterioration to SAM at 12 weeks**						
Lelijveld 2021 (1)	78	317	126	393	100.0%	0.77 [0.60, 0.98]
**Subtotal (95% CI)**		**317**		**393**	**100.0%**	**0.77 [0.60, 0.98]**
Total events	78		126			
Heterogeneity: Not applicable						
Test for overall effect: Z = 2.16 (P = 0.03)						
**1.2.2 Deterioration to SAM at 24 weeks**						
Lelijveld 2021 (2)	127	317	147	393	100.0%	1.07 [0.89, 1.29]
**Subtotal (95% CI)**		**317**		**393**	**100.0%**	**1.07 [0.89, 1.29]**
Total events	127		147			
Heterogeneity: Not applicable						
Test for overall effect: Z = 0.72 (P = 0.47)						

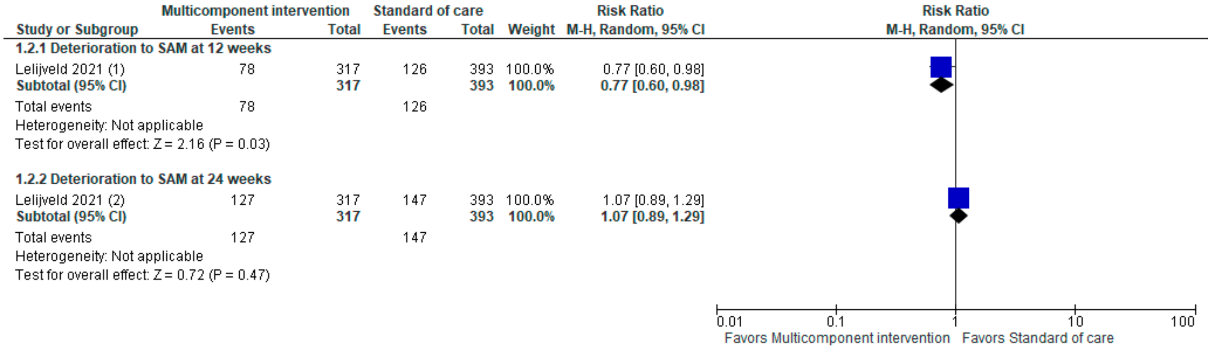

Footnotes

(1) High risk MAM children at 12 weeks, SAM defined as MUAC <11.5cm

(2) High risk MAM children at 24 weeks, SAM defined as MUAC <11.5cm

Supporting Figure S17: Forest plot of multicomponent intervention compared to non-food-based or none - Outcome: Weight for height z-score.

Study or Subgroup	Multicomponent intervention Mean	SD	Total	Standard of care or none Mean	SD	Total	Weight	Mean Difference IV, Random, 95% CI
**1.3.1 WHZ at 12 weeks**								
Lelijveld 2021	-1.54	0.92	317	-1.56	0.93	393	100.0%	0.02 [-0.12, 0.16]
**Subtotal (95% CI)**			**317**			**393**	**100.0%**	**0.02 [-0.12, 0.16]**
Heterogeneity: Not applicable								
Test for overall effect: Z = 0.29 (P = 0.77)								
**1.3.2 WHZ at 24 groups**								
Lelijveld 2021	-1.46	1.03	317	-1.55	0.98	393	100.0%	0.09 [-0.06, 0.24]
**Subtotal (95% CI)**			**317**			**393**	**100.0%**	**0.09 [-0.06, 0.24]**
Heterogeneity: Not applicable								
Test for overall effect: Z = 1.18 (P = 0.24)								

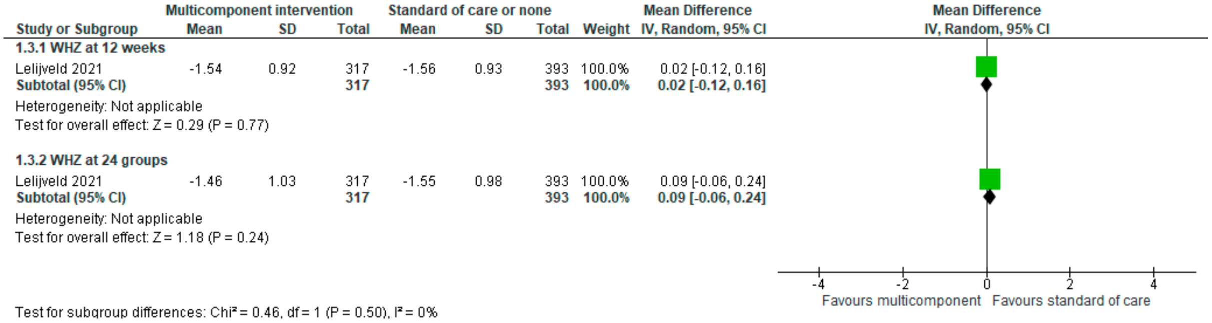

Test for subgroup differences: Chi² = 0.46, df = 1 (P = 0.50), I² = 0%

Supporting Figure S18: Forest plot of multicomponent intervention compared to non-food-based or none - Outcome: Weight for age z-score.

	Multicomponent intervention			Standard of care				Mean Difference
Study or Subgroup	Mean	SD	Total	Mean	SD	Total	Weight	IV, Random, 95% CI
**1.4.1 WAZ at 12 weeks**								
Lelijveld 2021	-2.78	0.96	317	-2.88	1.02	393	100.0%	0.10 [-0.05, 0.25]
**Subtotal (95% CI)**			**317**			**393**	**100.0%**	**0.10 [-0.05, 0.25]**
Heterogeneity: Not applicable								
Test for overall effect: Z = 1.34 (P = 0.18)								
**1.4.2 WAZ at 24 weeks**								
Lelijveld 2021	-2.72	1.08	317	-2.86	1.02	393	100.0%	0.14 [-0.02, 0.30]
**Subtotal (95% CI)**			**317**			**393**	**100.0%**	**0.14 [-0.02, 0.30]**
Heterogeneity: Not applicable								
Test for overall effect: Z = 1.76 (P = 0.08)								

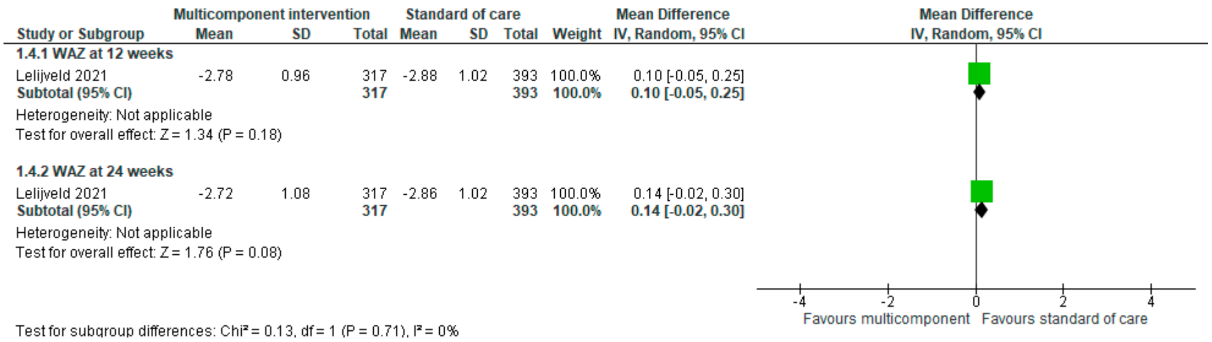

Test for subgroup differences: Chi² = 0.13, df = 1 (P = 0.71), I² = 0%

Supporting Figure S19: Forest plot of multicomponent intervention compared to non-food-based or none - Outcome: Height for age z-score.

	Multicomponent intervention			Standard of care or none				Mean Difference
Study or Subgroup	Mean	SD	Total	Mean	SD	Total	Weight	IV, Random, 95% CI
**1.5.1 HAZ at 12 weeks**								
Lelijveld 2021	-3.12	1.18	317	-3.28	1.28	393	100.0%	0.16 [-0.02, 0.34]
**Subtotal (95% CI)**			**317**			**393**	**100.0%**	**0.16 [-0.02, 0.34]**
Heterogeneity: Not applicable								
Test for overall effect: Z = 1.73 (P = 0.08)								
**1.5.2 HAZ at 24 weeks**								
Lelijveld 2021	-3.2	1.21	317	-3.31	1.26	393	100.0%	0.11 [-0.07, 0.29]
**Subtotal (95% CI)**			**317**			**393**	**100.0%**	**0.11 [-0.07, 0.29]**
Heterogeneity: Not applicable								
Test for overall effect: Z = 1.18 (P = 0.24)								

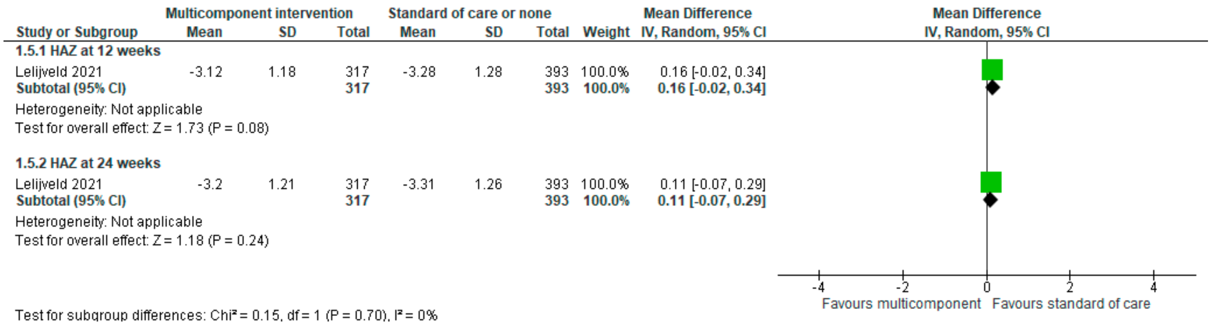

Test for subgroup differences: Chi² = 0.15, df = 1 (P = 0.70), I² = 0%

Supporting Figure S20: Forest plot of multicomponent intervention compared to non-food-based or none - Outcome: MUAC gain.

	Multicomponent intervention			Standard of care or none				Mean Difference
Study or Subgroup	Mean	SD	Total	Mean	SD	Total	Weight	IV, Random, 95% CI
**1.7.1 MUAC gain from enrollment to 12 weeks (in cm)**								
Lelijveld 2021	0.61	0.81	317	0.56	0.84	393	58.6%	0.05 [-0.07, 0.17]
**Subtotal (95% CI)**			**317**			**393**	**58.6%**	**0.05 [-0.07, 0.17]**
Heterogeneity: Not applicable								
Test for overall effect: Z = 0.80 (P = 0.42)								
**1.7.2 MUAC gain from enrollment to 24 weeks (in cm)**								
Lelijveld 2021	0.88	0.97	317	0.78	0.99	393	41.4%	0.10 [-0.04, 0.24]
**Subtotal (95% CI)**			**317**			**393**	**41.4%**	**0.10 [-0.04, 0.24]**
Heterogeneity: Not applicable								
Test for overall effect: Z = 1.35 (P = 0.18)								
**Total (95% CI)**			**634**			**786**	**100.0%**	**0.07 [-0.02, 0.16]**
Heterogeneity: Tau² = 0.00; Chi² = 0.27, df = 1 (P = 0.60); I² = 0%								
Test for overall effect: Z = 1.49 (P = 0.14)								
Test for subgroup differences: Chi² = 0.27, df = 1 (P = 0.60), I² = 0%								

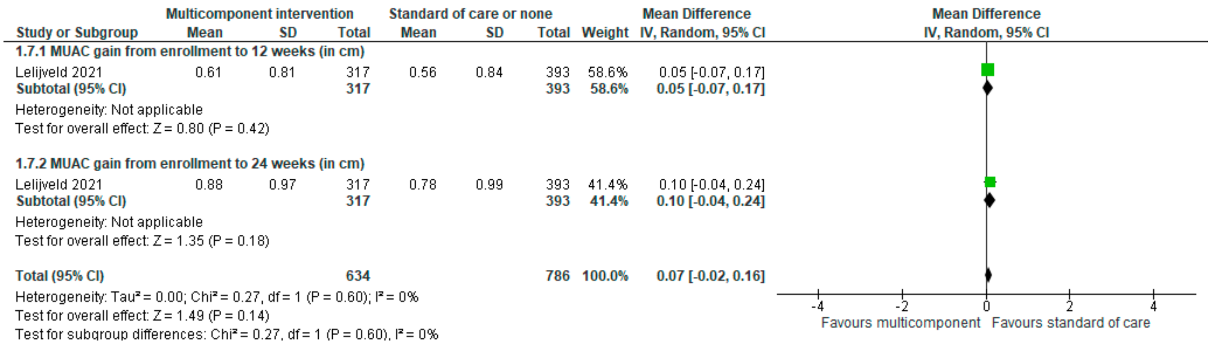

Supporting Figure S21: Forest plot of multicomponent intervention compared to non-food-based or none - Outcome: Weight gain.

	Multicomponent intervention			Standard of Care				Mean Difference
Study or Subgroup	Mean	SD	Total	Mean	SD	Total	Weight	IV, Random, 95% CI
**1.8.1 Average weight gain at 12 weeks (g/kg/day)**								
Lelijveld 2021	1.29	0.98	317	1.16	1.05	393	100.0%	0.13 [-0.02, 0.28]
**Subtotal (95% CI)**			**317**			**393**	**100.0%**	**0.13 [-0.02, 0.28]**
Heterogeneity: Not applicable								
Test for overall effect: Z = 1.70 (P = 0.09)								
**1.8.2 Average weight gain at 24 weeks (g/kg/day)**								
Lelijveld 2021	1.1	0.68	317	0.98	0.64	393	100.0%	0.12 [0.02, 0.22]
**Subtotal (95% CI)**			**317**			**393**	**100.0%**	**0.12 [0.02, 0.22]**
Heterogeneity: Not applicable								
Test for overall effect: Z = 2.40 (P = 0.02)								

Test for subgroup differences: Chi² = 0.01, df = 1 (P = 0.91), I² = 0%

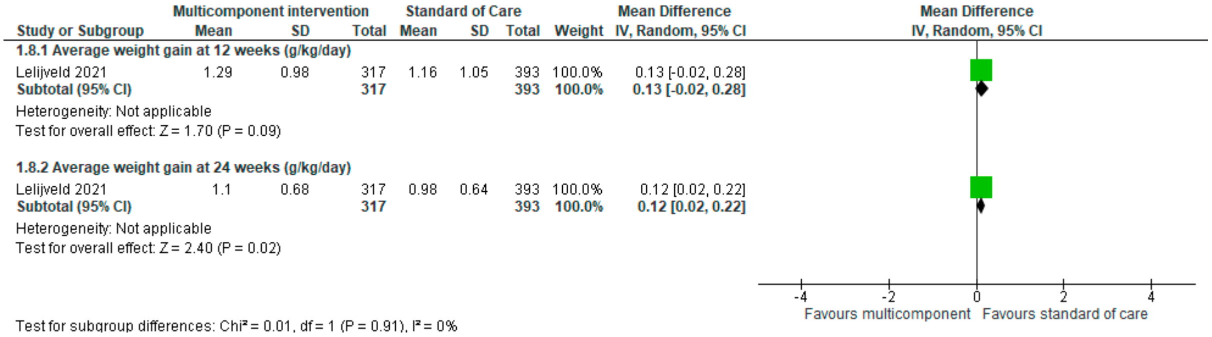

Supporting Figure S22: Forest plot of multicomponent intervention compared to non-food-based or none - Outcome: Non-response.

	Multicomponent intervention		Standard of care			Risk Ratio
Study or Subgroup	Events	Total	Events	Total	Weight	M-H, Random, 95% CI
**1.9.1 Non-response at 12 weeks**						
Lelijveld 2021 (1)	89	317	86	393	100.0%	1.28 [0.99, 1.66]
**Subtotal (95% CI)**		**317**		**393**	**100.0%**	**1.28 [0.99, 1.66]**
Total events	89		86			
Heterogeneity: Not applicable						
Test for overall effect: Z = 1.90 (P = 0.06)						
**1.9.2 Non-response at 24 weeks**						
Lelijveld 2021 (2)	32	317	30	393	100.0%	1.32 [0.82, 2.13]
**Subtotal (95% CI)**		**317**		**393**	**100.0%**	**1.32 [0.82, 2.13]**
Total events	32		30			
Heterogeneity: Not applicable						
Test for overall effect: Z = 1.15 (P = 0.25)						

Test for subgroup differences: Chi² = 0.01, df = 1 (P = 0.91), I² = 0%

Footnotes
(1) Remained with MAM
(2) Remained with MAM at 24 weeks

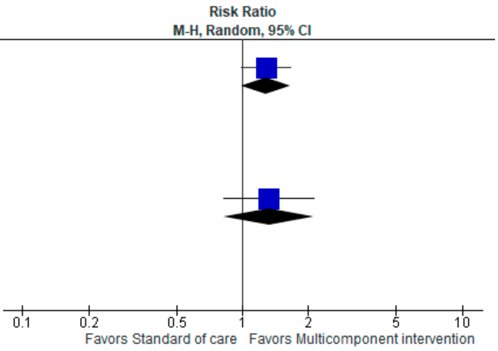

Supporting Figure S23: Forest plot of multicomponent intervention compared to non-food-based or none - Outcome: Mortality.

	Multicomponent intervention		Standard of care			Risk Ratio
Study or Subgroup	Events	Total	Events	Total	Weight	M-H, Random, 95% CI
**1.10.1 Mortality at 12 weeks**						
Lelijveld 2021	4	317	12	393	100.0%	0.41 [0.13, 1.27]
**Subtotal (95% CI)**		**317**		**393**	**100.0%**	**0.41 [0.13, 1.27]**
Total events	4		12			
Heterogeneity: Not applicable						
Test for overall effect: Z = 1.54 (P = 0.12)						
**1.10.2 Mortality at 24 weeks**						
Lelijveld 2021	9	317	20	393	100.0%	0.56 [0.26, 1.21]
**Subtotal (95% CI)**		**317**		**393**	**100.0%**	**0.56 [0.26, 1.21]**
Total events	9		20			
Heterogeneity: Not applicable						
Test for overall effect: Z = 1.48 (P = 0.14)						

Test for subgroup differences: Chi² = 0.19, df = 1 (P = 0.67), I² = 0%

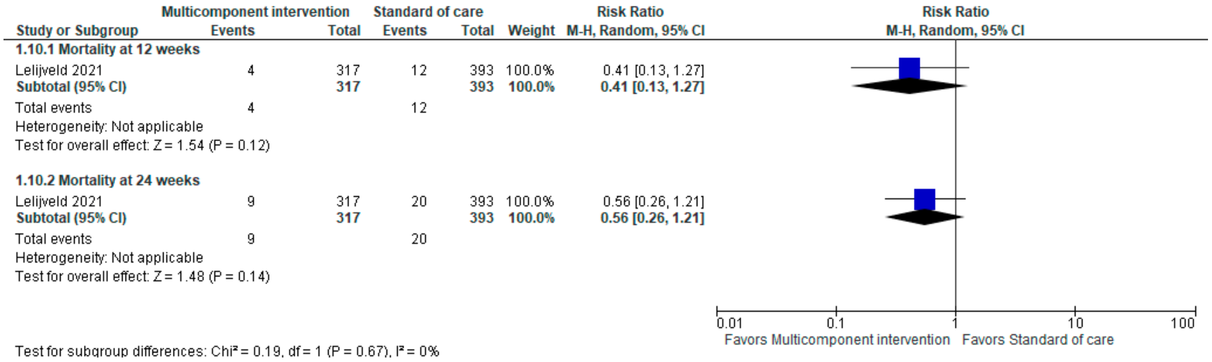

Supplement: Supplementary file 1 [file nutrients-15-03781-s001.zip › Annex 2. Supporting Figures.pdf]
